# Real‐world persistence and dose titration of GLP‐1 receptor agonists in type 2 diabetes: A UK population‐based cohort study by obesity and cardiovascular disease status

**DOI:** 10.1111/dom.70535

**Published:** 2026-02-17

**Authors:** Franziska S. Ulrich, Nicola Napoli, Morten Frost Nielsen, Andrea M. Burden

**Affiliations:** ^1^ Institute of Pharmaceutical Sciences ETH Zurich Zurich Switzerland; ^2^ Unit of Endocrinology and Diabetes, Campus Bio‐Medico University of Rome Rome Italy; ^3^ Unit of Metabolic Bone and Thyroid Disorders, Fondazione Policlinico Universitario Campus Bio‐Medico University of Rome Rome Italy; ^4^ Division of Bone and Mineral Diseases Washington University St Louis Missouri USA; ^5^ Department of Endocrinology Odense University Hospital Odense Denmark; ^6^ Steno Diabetes Centre Odense Odense University Hospital Odense Denmark; ^7^ Division of Clinical Immunology and Rheumatology, Heersink School of Medicine University of Alabama at Birmingham Birmingham Alabama USA

**Keywords:** Type 2 diabetes, GLP‐1 receptor agonist, obesity, cardiovascular disease, primary care, treatment discontinuation, dose titration, United Kingdom, real‐world evidence, glucose‐lowering therapy, drug utilisation

## Abstract

**Aims:**

Real‐world medication use varies across clinical trial and healthcare settings; therefore, we evaluated GLP‐1 receptor agonist (GLP‐1RA) persistence and dose titration among adults with type 2 diabetes in UK primary care, stratified by agent, obesity status, cardiovascular disease (CVD) history, and sex assigned at birth.

**Materials and methods:**

Adults with type 2 diabetes initiating GLP‐1RAs between 1 March 2018 and 30 June 2023, with follow‐up ≥1‐year, were identified using the IQVIA Medical Research Data (IMRD) incorporating data from THIN, A Cegedim Database. The absolute 1‐year risk of discontinuation was estimated using the Aalen‐Johansen estimator, while multivariable cause‐specific Cox models assessed associations with patient characteristics. Dosing trajectories were characterised across individual prescriptions during the first therapy year.

**Results:**

Among 8200 GLP‐1RA initiators (46.5% dulaglutide, 38.7% semaglutide), the 1‐year discontinuation risk was 41.1% (95% CI 40.1–42.2), higher among those without obesity (BMI <30 kg/m^2^: 49.2%, 46.5–52.0) versus with BMI ≥30 kg/m^2^ (BMI 30–<35/≥35 kg/m^2^: 40.4%/37.2%, 38.4–42.4/35.4–39.1). Discontinuation was lower with dulaglutide (36.9%, 35.4–38.4) than with subcutaneous/oral semaglutide (46.4%/55.8%, 44.4–48.4/52.3–59.4). Only 58% of subcutaneous/oral semaglutide initiators reached recommended maintenance doses after 4 weeks, while 21% remained on starting doses. At prescription 10, most common doses were 0.5/1 mg subcutaneous semaglutide (44.0%/48.6%), 7/14 mg oral semaglutide (50.2%/36.6%), and 0.75/1.5/3 mg dulaglutide (20.4%/71.5%/6.7%). Faster dose escalation occurred with higher BMI and subcutaneous semaglutide (1 mg at prescription 5: 20.8%/25.9%/33.2% with BMI <30/30–<35/≥35 kg/m^2^). Additional analyses (e.g., by CVD history) revealed no further heterogeneity.

**Conclusions:**

In UK primary care, GLP‐1RA persistence was suboptimal and dose escalation was frequently delayed across all patient stratifications, including individuals with established CVD.

## 1 INTRODUCTION

Type 2 diabetes is a chronic and progressive condition affecting over 500 million patients globally^1^ and is associated with both microvascular and macrovascular complications, including cardiovascular disease (CVD).^2^, ^3^ The American Diabetes Association (ADA) and the European Association for the Study of Diabetes (EASD) endorse a patient‐centred approach to glycaemic management, recommending a glucagon‐like peptide‐1 receptor agonist (GLP‐1RA) or a sodium‐glucose cotransporter‐2 inhibitor (SGLT‐2i) for individuals with type 2 diabetes who have a high risk of or established CVD, heart failure, chronic kidney disease (CKD), or obesity.^4^, ^5^ In contrast, the National Institute for Health and Care Excellence (NICE) guideline NG28 from the United Kingdom (UK) only recommends GLP‐1RAs if triple oral therapy was ineffective, not tolerated, or contraindicated. Moreover, use is restricted to individuals with a body mass index (BMI) ≥35 kg/m^2^ with obesity‐related complications, or to those with BMI <35 kg/m^2^ for whom insulin would pose occupational difficulties.^6^

Despite evidence from clinical trials demonstrating benefits in glycaemic control, weight loss, and cardiorenal outcomes,^7^, ^8^, ^9^, ^10^ real‐world adherence to GLP‐1RA therapy remains suboptimal in patients with type 2 diabetes.^11^, ^12^, ^13^ Suboptimal adherence and low rates of continued medication use have been linked to poorer glycaemic control, increased healthcare utilization, and higher costs.^11^, ^12^, ^13^ In addition to treatment persistence, adherence to dose escalation protocols is required for optimal efficacy, as specified in the Summary of Product Characteristics (SPC).^14^, ^15^, ^16^ Nevertheless, previous observational studies have identified that the majority of patients do not reach recommended maintenance doses prior to discontinuation.^17^, ^18^, ^19^ However, to date, evidence on how GLP‐1RA dose regimens are followed in UK clinical practice remains limited.

To address this research gap, we used a large, UK‐representative primary care database to characterize GLP‐1RA treatment courses during the first year after initiation between 2018 and 2022 in adults with type 2 diabetes. Specifically, we (i) quantified GLP‐1RA discontinuation over 1 year; (ii) described agent‐specific dosing trajectories and adherence to SPC‐recommended dose‐escalation schedules; and (iii) examined whether persistence and dose titration differed by BMI category, CVD history, and sex assigned at birth. By embedding these patterns in the context of contemporary NICE guidance and international cardiovascular prevention guidelines,^4^, ^5^, ^6^ we sought to identify potential evidence‐practice gaps and inefficiencies in the use of GLP‐1RAs in UK care.

## 2 MATERIALS AND METHODS

### 2.1 Data source

This UK population‐based cohort study leveraged the IQVIA Medical Research Data (IMRD) incorporating data from THIN, A Cegedim Database. As of November 2022, the IMRD includes electronic medical records (EMRs) from over 25 million patients registered with general practitioner (GP) practices throughout the UK.^20^ The IMRD population is broadly representative of the general UK population and comparable to other widely used UK primary care databases, including the Clinical Practice Research Datalink (CPRD) GOLD.^21^, ^22^ The IMRD captures longitudinal information on patients' demographics, clinical diagnoses and procedures (both recorded using the Read code classification), laboratory tests (e.g., glycated haemoglobin [HbA1c]), medication prescriptions, as well as lifestyle indicators (e.g., smoking status).^20^, ^21^

### 2.2 Study design and population

Using the IMRD, we identified a cohort of adults (aged ≥18 years) who initiated GLP‐1RA therapy (see Table S1, Supporting Information for exposure definition) between 1 March 2018 (based on the market authorization of the most recently approved GLP‐1RA, semaglutide, by the European Medicines Agency)^14^ and 30 June 2023 (end of data availability).

Cohort entry was defined as the date of the first GLP‐1RA prescription. To be included, individuals had a diagnosis of type 2 diabetes at or before cohort entry, at least 1 year of continuous registration with their GP practice prior to cohort entry, and a minimum of 1 year of follow‐up in the IMRD (i.e., GLP‐1RA start prior to 30 June 2022). We excluded patients with a prior diagnosis of type 1 diabetes, gestational diabetes, or polycystic ovary syndrome before or at cohort entry, and those with insulin as first‐line glucose‐lowering therapy.^23^ Moreover, patients were excluded if they began treatment with more than one GLP‐1RA agent or initiated a fixed‐dose combination therapy with insulin at cohort entry (see Table S1).

Eligible patients were followed from cohort entry until the earliest occurrence of death, transfer out of the GP practice, end of data availability (30 June 2023), or end of follow‐up (defined as 1‐year plus a 120‐day extension to allow for the assessment of discontinuation patterns across the full 1‐year study period).^19^

### 2.3 Covariates

Patient characteristics, including demographics (e.g., age, sex assigned at birth), lifestyle indicators (e.g., smoking status), laboratory and vital‐sign measurements (e.g., BMI, HbA1c), previous and concomitant use of medications (e.g., SGLT‐2is, statins), as well as lifetime history of comorbidities (e.g., CVD, cancer) were assessed during the year before or at cohort entry (Tables S1–S3).

### 2.4 Definition of exposure

Prescriptions for glucose‐lowering agents recorded between 1 January 2000 and cohort entry were classified as individual GLP‐1RAs, metformin, sulfonylureas, SGLT‐2is, DPP‐4is, thiazolidinediones, other oral glucose‐lowering agents, and insulin (Tables S1 and S2). Prescriptions for combination products were disaggregated into their respective individual components. For GLP‐1RAs, we defined the route of administration (i.e., subcutaneous injection or oral tablet) and dose for each prescription based on Table S1.

### 2.5 Outcomes

The co‐primary outcomes of interest were: (1) the discontinuation of GLP‐1RA therapy within the first year following treatment initiation; and (2) the dose titration trajectories of individual GLP‐1RA agents across the first 10 prescriptions, which were assumed to reflect treatment patterns during the first year of therapy.

GLP‐1RA discontinuation was defined as a gap of >90 days between two consecutive prescriptions.^17^ The discontinuation date was assigned as the day following the end of coverage of the last prescription (assuming a standard 30‐day supply per prescription and allowing for a maximum permissible gap of 60 days). Switching between GLP‐1RA agents was not considered discontinuation. A secondary analysis stratified discontinuation by GLP‐1RA agent, where discontinuation was defined as either a gap of >90 days between subsequent prescriptions or a switch to a different GLP‐1RA agent. In a sensitivity analysis, the permissible gap between prescriptions was varied to >60 days and >120 days.

Discontinuation was evaluated overall, as well as stratified by: (1) BMI categories, defined as <30, ≥30 to <35, and ≥35 kg/m^2^ based on the most recent BMI measurement recorded within the year before or at cohort entry (patients with no recent BMI measurement available were grouped as “unknown”); (2) index GLP‐1RA agent (i.e., semaglutide overall and by formulation [subcutaneous or oral], dulaglutide, liraglutide, exenatide, and lixisenatide); (3) history of CVD (i.e., having a diagnosis of myocardial infarction, stable or unstable angina, coronary atherosclerosis, transient ischaemic attack or stroke, coronary procedures, or heart failure recorded prior to or at cohort entry); and (4) sex assigned at birth.

Dose titration trajectories were assessed for patients initiating injectable semaglutide, oral semaglutide, or dulaglutide, for which detailed dosing information was available in the IMRD (agents such as liraglutide were not considered for dosing analyses due to the use of variable‐dose injection pens that precluded dose ascertainment; see Table S1). Furthermore, dosing patterns were also stratified by BMI category, CVD history, and sex assigned at birth.

### 2.6 Statistics

Baseline characteristics were described as counts with percentages and medians with 25th and 75th percentiles (interquartile range [IQR]), stratified by GLP‐1RA agent.

Cause‐specific cumulative incidence curves and the absolute risk of GLP‐1RA discontinuation were derived using the Aalen‐Johansen estimator to provide unadjusted estimates, with death as a competing risk.

We used Sankey diagrams to visualize dose titration trajectories across the first 10 GLP‐1RA prescriptions of patients who initiated semaglutide (oral or subcutaneous) and dulaglutide.^17^, ^24^ We illustrate counts with percentages receiving a specific dose, along with the median time since initiation and corresponding IQR at each prescription number (e.g., first, second, etc.). All analyses were stratified by BMI category, CVD history, and sex assigned at birth.

In supplementary analyses, we assessed factors (i.e., index GLP‐1RA agent initiated, BMI category, CVD history, and sex assigned at birth) associated with the risk of GLP‐1RA discontinuation using cause‐specific Cox proportional hazards regression analyses, with censoring at the date of death or transfer out of the GP practice. The multivariable Cox model was restricted to patients with a recent BMI measurement and was adjusted for variables specified in Table S3.

Results of statistical analyses were estimated with corresponding two‐sided 95% confidence intervals (95% CIs), while *p*‐values were determined using *α* = 0.05. Individual counts with <7 events were suppressed to prevent person identification. All analyses were conducted employing the statistical software R (version 4.2.2; R Core Team, 2022) and SAS (version 9.4; SAS Institute Inc., 2016).

## 3 RESULTS

We identified 8200 adults with type 2 diabetes initiating GLP‐1RA therapy in UK primary care between 2018 and 2022 (Figure S1), receiving a total of 164 833 GLP‐1RA prescriptions, with a median of 17 prescriptions per person (IQR 8–29) and a median interval of 28 days (IQR 25–35) between consecutive prescriptions. The most frequently initiated GLP‐1RA agents were dulaglutide (*n* = 3812; 46.5%) and semaglutide (*n* = 3173; 38.7%), followed by liraglutide (*n* = 974; 11.9%).

### 3.1 Characteristics of GLP‐1RA initiators

Baseline characteristics, presented overall and stratified by GLP‐1RA agent, are shown in Table 1 for semaglutide and dulaglutide initiators, and in Tables S4 and S5 for initiators of other GLP‐1RA agents.

**TABLE 1:** Characteristics of individuals with type 2 diabetes initiating glucagon‐like peptide‐1 (GLP‐1) receptor agonist therapy between 2018 and 2022, overall and by semaglutide and dulaglutide subgroups.

Characteristics	Overall (*n* = 8200)	Semaglutide (*n* = 3173)	Dulaglutide (*n* = 3812)
Age at initiation, years	60.7 [53.1, 68.6]	60.6 [52.7, 68.7]	61.0 [53.5, 69.0]
Female sex assigned at birth	3695 (45.1%)	1381 (43.5%)	1729 (45.4%)
Type 2 diabetes duration^a^, years	6.9 [3.4, 11.6]	6.8 [3.3, 11.5]	7.0 [3.4, 11.7]
Previous use of glucose‐lowering therapies^b^			
Metformin	7999 (97.5%)	3087 (97.3%)	3729 (97.8%)
Sulfonylureas	4830 (58.9%)	1712 (54.0%)	2317 (60.8%)
SGLT‐2 inhibitors	4728 (57.7%)	1895 (59.7%)	2211 (58.0%)
DPP‐4 inhibitors	4978 (60.7%)	1874 (59.1%)	2357 (61.8%)
Thiazolidinediones	1381 (16.8%)	495 (15.6%)	646 (16.9%)
Insulin	1159 (14.1%)	402 (12.7%)	554 (14.5%)
Other glucose‐lowering therapies	112 (1.4%)	38 (1.2%)	52 (1.4%)
Number of glucose‐lowering therapies ≥3	5730 (69.9%)	2118 (66.8%)	2730 (71.6%)
Concurrent use of glucose‐lowering therapies^c^			
Metformin	6965 (84.9%)	2721 (85.8%)	3236 (84.9%)
Sulfonylureas	3385 (41.3%)	1176 (37.1%)	1632 (42.8%)
SGLT‐2 inhibitors	3890 (47.4%)	1563 (49.3%)	1831 (48.0%)
DPP‐4 inhibitors	3811 (46.5%)	1450 (45.7%)	1804 (47.3%)
Thiazolidinediones	386 (4.7%)	142 (4.5%)	181 (4.7%)
Insulin	1043 (12.7%)	353 (11.1%)	505 (13.2%)
Other glucose‐lowering therapies	22 (0.3%)	<7	13 (0.3%)
Number of glucose‐lowering therapies ≥3	3891 (47.5%)	1430 (45.1%)	1876 (49.2%)
Comorbidities^d^			
Cardiovascular disease	1565 (19.1%)	569 (17.9%)	738 (19.4%)
Myocardial infarction	554 (6.8%)	214 (6.7%)	243 (6.4%)
Stroke	315 (3.8%)	110 (3.5%)	165 (4.3%)
Heart failure	319 (3.9%)	118 (3.7%)	146 (3.8%)
Hypertension	5293 (64.5%)	2027 (63.9%)	2482 (65.1%)
Dyslipidaemia	1608 (19.6%)	622 (19.6%)	762 (20.0%)
Sleep apnea	390 (4.8%)	150 (4.7%)	175 (4.6%)
Asthma	1863 (22.7%)	727 (22.9%)	893 (23.4%)
Chronic obstructive pulmonary disease	2143 (26.1%)	819 (25.8%)	1025 (26.9%)
Chronic kidney disease	1383 (16.9%)	511 (16.1%)	654 (17.2%)
Osteoarthritis	1984 (24.2%)	761 (24.0%)	944 (24.8%)
Depression	3378 (41.2%)	1287 (40.6%)	1576 (41.3%)
Cancer	707 (8.6%)	270 (8.5%)	345 (9.1%)
Comedications^e^			
Statins	6395 (78.0%)	2452 (77.3%)	3006 (78.9%)
Angiotensin converting enzyme inhibitors	3815 (46.5%)	1466 (46.2%)	1792 (47.0%)
Angiotensin II receptor blockers	1557 (19.0%)	592 (18.7%)	731 (19.2%)
Diuretics	2283 (27.8%)	831 (26.2%)	1075 (28.2%)
Calcium channel blockers	199 (2.4%)	72 (2.3%)	93 (2.4%)
Beta‐blockers	2296 (28.0%)	870 (27.4%)	1073 (28.1%)
Acetylsalicylic acid	1773 (21.6%)	650 (20.5%)	802 (21.0%)
Other antiplatelets	686 (8.4%)	245 (7.7%)	338 (8.9%)
Anticoagulants	723 (8.8%)	264 (8.3%)	360 (9.4%)
Laboratory and vitalsign measurements^f^			
BMI, kg/m^2^	34.0 [30.6, 38.1]	34.0 [30.5, 38.1]	33.8 [30.5, 37.9]
BMI <30 kg/m^2^	1282 (15.2%)	508 (16.0%)	619 (16.3%)
BMI 30–<35 kg/m^2^	2251 (27.5%)	847 (26.7%)	1076 (28.2%)
BMI ≥35 kg/m^2^	2712 (33.1%)	1027 (32.4%)	1231 (32.3%)
BMI missing	1955 (23.8%)	791 (24.9%)	886 (23.2%)
HbA1c, %	8.2 [7.6, 8.6]	8.1 [7.6, 8.6]	8.3 [7.7, 8.6]
eGFR, mL/min per 1.73 m^2^	97.8 [80.1, 107.5]	98.2 [80.2, 107.6]	97.1 [79.8, 106.7]
Systolic blood pressure, mmHg	132.0 [124.0, 140.0]	134.0 [124.0, 141.0]	132.0 [123.0, 140.0]
Diastolic blood pressure, mmHg	79.0 [72.0, 84.0]	80.0 [72.0, 84.0]	79.0 [71.0, 84.0]
Serum cholesterol, mmol/L	4.3 [3.6, 5.1]	4.3 [3.6, 5.1]	4.3 [3.6, 5.1]
Lifestyle factors^g^			
Markers of current smoking	1143 (13.9%)	425 (13.4%)	552 (14.5%)

*Note*: Data presented in *n* (%) or median [IQR]. Counts <7 are suppressed to prevent person identification.

Abbreviations: BMI, body mass index; DPP‐4, dipeptidyl peptidase‐4; eGFR, estimated glomerular filtration rate; HbA1c, glycated haemoglobin; SGLT‐2, sodium‐glucose co‐transporter‐2.

^a^ Time (years) between the first non‐insulin glucose‐lowering therapy initiated and GLP‐1 receptor agonist initiation.

^b^ Defined as having at least one prescription for a glucose‐lowering therapy specified in Table S2 before GLP‐1 receptor agonist initiation.

^c^ Defined as having at least one prescription for a glucose‐lowering therapy specified in Table S2 within the 180 days before or at GLP‐1 receptor agonist initiation.

^d^ Diagnosis recorded before or at GLP‐1 receptor agonist initiation.

^e^ Defined as having at least one prescription within the 365 days before or at GLP‐1 receptor agonist initiation.

^f^ Most recent measurement recorded within the 365 days before or at GLP‐1 receptor agonist initiation. The proportions of missing values were 2.4–3.8% for eGFR, 6.8–10.3% for blood pressure (systolic and diastolic), 9.4–12.1% for serum cholesterol, and 38.8–39.8% for HbA1c.

^g^ Most recent record before or at GLP‐1 receptor agonist initiation.

Overall, 45.1% were female, with a median age of 60.7 years (IQR 53.1–68.6), HbA1c of 8.2% (IQR 7.6–8.6), and type 2 diabetes duration of 6.9 years (IQR 3.4–11.6). Most individuals (69.9%) had prior exposure to ≥3 glucose‐lowering therapies. The most commonly co‐prescribed glucose‐lowering therapies in the 180 days before or at GLP‐1RA initiation were metformin (84.9%), SGLT‐2is (47.4%), DPP‐4is (46.4%), and sulfonylureas (41.3%). Among individuals with a recent BMI measurement available (*n* = 6245), 79.5% met the World Health Organization (WHO) criteria for obesity (BMI ≥30 kg/m^2^),^25^ and 43.4% had a BMI ≥35 kg/m^2^. Common obesity‐ and type 2 diabetes‐related comorbidities included CVD (19.1%), hypertension (64.5%), dyslipidaemia (19.6%), and CKD (16.9%).

The characteristics of individuals initiating dulaglutide and semaglutide were generally comparable to the overall cohort (Tables 1 and S5).

### 3.2 Discontinuation patterns

**FIGURE 1:**
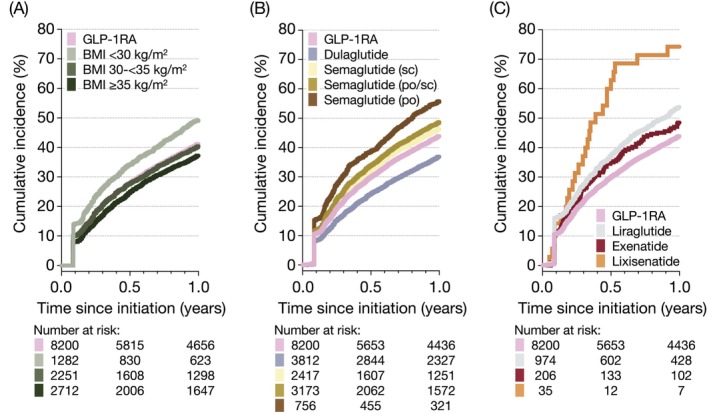
Cumulative incidence of glucagon‐like peptide‐1 (GLP‐1) receptor agonist therapy discontinuation over 1 year. (A) GLP‐1RA discontinuation (defined as a >90‐day prescription gap), stratified by body mass index (BMI); (B and C) GLP‐1RA discontinuation (defined as a >90‐day prescription gap or agent switch), stratified by index GLP‐1RA agent. See Tables S6–S8 for more details on GLP‐1RA therapy discontinuation. The graph was created using GraphPad Prism version 10.2.0 [335].

Overall, the absolute 1‐year risk of GLP‐1RA therapy discontinuation was 41.1% (95% CI 40.1–42.2; Figure 1A and Table S6). Discontinuation was most frequently observed during the first few months following treatment initiation, with the absolute 6‐month risk estimated at 27.9% (95% CI 27.0–28.9; Figure 1A and Table S6). Prespecified sensitivity analyses were consistent with overall results (Table S6).

The absolute risk of discontinuing GLP‐1RA therapy varied by BMI categories (Figure 1A and Table S7). The 1‐year risk was highest among patients with BMI <30 kg/m^2^ (49.2%, 95% CI 46.5–52.0) compared to those with BMI 30–<35 kg/m^2^ (40.4%, 95% CI 38.4–42.4) and BMI ≥35 kg/m^2^ (37.2%, 95% CI 35.4–39.1). No differences were observed when stratified by CVD history or sex assigned at birth (Table S7), with discontinuation risks of approximately 41–42% in all groups.

Discontinuation differed by the specific GLP‐1RA agent initiated (Figure 1B,C and Table S8). At 1 year, the absolute risk of discontinuation was lowest among those initiating dulaglutide (36.9%, 95% CI 35.4–38.4), followed by exenatide (48.5%, 95% CI 41.7–55.4), semaglutide (48.7%, 95% CI 46.9–50.4), and liraglutide (53.8%, 95% CI 50.7–56.9). The absolute 1‐year risk of discontinuing semaglutide was slightly higher among individuals initiating the oral formulation (55.8%, 95% CI 52.3–59.4) compared to those starting subcutaneous semaglutide (46.4%, 95% CI 44.4–48.4). Switching to a different GLP‐1RA agent within the first year of treatment was most frequently observed among exenatide (12.1%) and liraglutide (9.4%) initiators, while being uncommon (<4%) among those who started on dulaglutide or semaglutide.

### 3.3 Dosing trajectories

#### 3.3.1 Semaglutide dose titration

**FIGURE 2:**
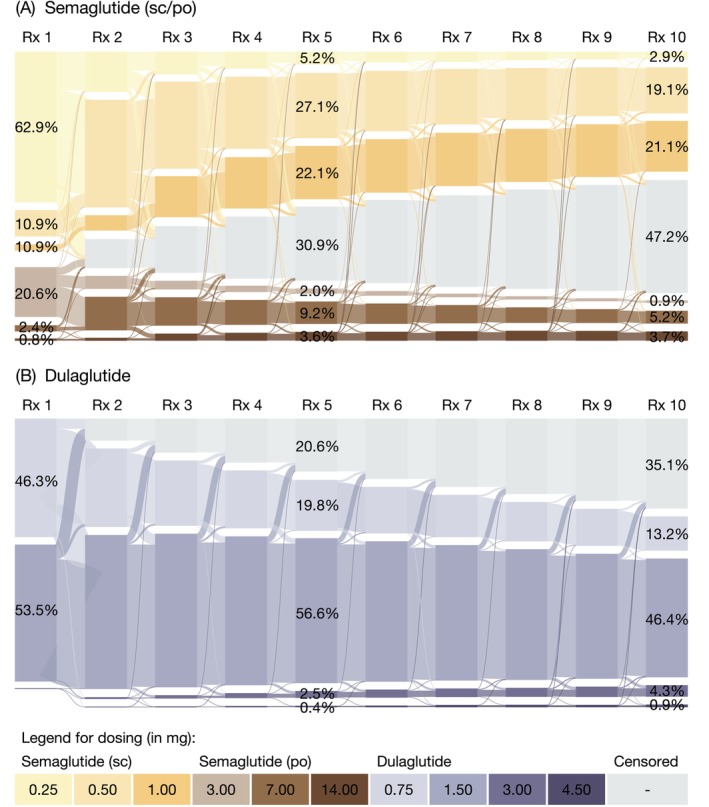
Sankey diagram illustrating dosing trajectories of (A) semaglutide (subcutaneous [sc] or oral tablet [po]) and (B) dulaglutide from the first to the 10th prescription (Rx). Values indicate the percentage of individuals receiving each dose at each prescription number (full details in Tables S9 and S13). The graph was generated using the R package networkD3 (Allaire et al.^24^).

The Sankey diagram shown in Figure 2A illustrates the dosing trajectories of semaglutide, with supporting values in Table S9. Among semaglutide initiators (*n* = 3173), 76.2% (*n* = 2417) started with the subcutaneous injection formulation (82.6% at the 0.25 mg dose) and 23.8% (*n* = 756) initiated the oral tablet formulation (86.6% at 3 mg dose). Discontinuation after the first prescription occurred in 10.9% of subcutaneous initiators compared to 15.5% of those initiating the oral formulation. By the second prescription, 58.8% of individuals initiating subcutaneous semaglutide used the 0.5 mg dose, while 58.2% of those initiating oral semaglutide progressed to the 7 mg dose. At the third prescription, 22.5% of subcutaneous initiators further escalated to 1 mg, and 11.9% of oral initiators to 14 mg.

The median time from semaglutide initiation to the tenth prescription was 36.4 weeks (IQR 34.0–41.0) for subcutaneous and 38.1 weeks (IQR 35.0–50.6) for oral initiators, with 56.2% (*n* = 1359) and 41.9% (*n* = 317), respectively, receiving a tenth semaglutide prescription. Among those who received a tenth prescription, the most common doses were 0.5 mg (44.0%) and 1 mg (48.6%) for subcutaneous initiators, and 7 mg (50.2%) and 14 mg (36.6%) for oral initiators. Cross‐formulation switching was rare, with 0.3% of subcutaneous and 2.1% of oral semaglutide initiators receiving prescriptions for the other formulation at the tenth prescription.

Semaglutide dosing trajectories varied by BMI categories (Table S10), with patients with higher BMI reaching higher doses more quickly. By the fifth prescription, the proportions reaching 1 mg subcutaneous or 14 mg oral formulations were as follows: 33.2% or 18.0% for BMI ≥35 kg/m^2^, 25.9% or 14.8% for BMI 30–<35 kg/m^2^, and 20.8% or 12.7% for BMI <30 kg/m^2^, respectively. Moreover, individuals with higher BMI were more likely to persist and received a tenth prescription for both subcutaneous and oral formulations: 60.2% and 47.8% for BMI ≥35 kg/m^2^, 54.7% and 41.1% for BMI 30–<35 kg/m^2^, and 48.3% and 37.6% for BMI <30 kg/m^2^, respectively. When stratified by CVD history and sex assigned at birth, the results were consistent with the overall cohort, with no notable differences between strata observed (Tables S11 and S12).

#### 3.3.2 Dulaglutide dose titration

Figure 2B illustrates the Sankey diagram for the dosing trajectories of dulaglutide initiators (*n* = 3812), with supporting values in Table S13. The most common starting doses were 0.75 mg (*n* = 1765; 46.3%) and 1.5 mg (*n* = 2041; 53.5%; Figure 2B), while 8.5% discontinued after the first prescription. At the second and third prescriptions, the most common doses remained 0.75 mg (30.6% vs. 25.4%) and 1.5 mg (60.2% vs. 60.0%), with a small proportion escalating to 3 mg (0.6% vs. 1.2%). Of those who received a tenth prescription (*n* = 2474; 64.9%), most received 0.75 mg (20.4%), 1.5 mg (71.5%), or 3.0 mg (6.7%) doses, at a median of 35.9 weeks [IQR 33.9, 39.0] after initiation.

Similar to semaglutide, dulaglutide dosing trajectories varied by BMI (Table S13), with individuals in higher BMI categories reaching higher doses more quickly by the fifth prescription (e.g., for 3 mg: 2.8% with BMI ≥35 kg/m^2^, 2.0% for BMI 30–<35 kg/m^2^, and 1.3% for BMI <30 kg/m^2^). Additionally, individuals with higher BMI were more likely to receive a tenth prescription. No CVD history or sex‐specific differences were observed (Table S14).

### 3.4 Factors of GLP‐1RA discontinuation

Using the multivariable cause‐specific Cox model, restricted to the 6245 (76.2%) individuals with a recent BMI measurement, we confirmed above results (Table S15): Individuals with obesity (BMI 30–<35 kg/m^2^ or BMI ≥35 kg/m^2^) were less likely to discontinue GLP‐1RA therapy than those with BMI <30 kg/m^2^. While initiation with oral semaglutide increased the risk of discontinuation compared to the subcutaneous formulation, CVD and sex assigned at birth strata did not change discontinuation patterns.

## 4 DISCUSSION

In this UK population‐based cohort study of 8200 adults with type 2 diabetes initiating GLP‐1RAs in primary care, 41% discontinued therapy within the first year. Persistence differed between agents, being lower for oral semaglutide (55.8%) than for subcutaneous semaglutide (46.4%) and dulaglutide (36.9%). Beyond persistence, we found that dose titration frequently deviated from SPC‐recommended schedules: only around half of semaglutide initiators had escalated to recommended maintenance doses by the second prescription, and escalation to higher maintenance doses remained limited, particularly among dulaglutide initiators. Despite the cardio‐protective benefits of GLP‐1RAs, we did not observe differences in discontinuation or dosing patterns by CVD history. Patterns were more favourable among individuals with obesity, particularly those with BMI ≥35 kg/m^2^, who were less likely to discontinue therapy and more likely to reach higher doses.

Our finding that 41% of GLP‐1RA initiators discontinue therapy within 1‐year aligns with a previous UK‐based study by Weiss et al.^26^ and other international real‐world studies on GLP‐1RA utilization with discontinuation rates ranging from 21% to >50%.^19^, ^27^, ^28^, ^29^, ^30^ Regional and interstudy variability in discontinuation rates likely reflect differences in populations, data sources, healthcare systems, clinical guidelines, and reimbursement frameworks.^19^, ^26^, ^27^, ^28^, ^29^, ^30^ For instance, UK's NICE guidelines (NG28) for type 2 diabetes management recommend discontinuing GLP‐1RA therapy after 6‐months if a reduction of at least 1% in HbA1c and 3% in bodyweight was not achieved,^6^ which may partly account for the high early discontinuation rates observed. Additionally, early discontinuation may also reflect treatment cessation due to adverse events, including nausea and other gastrointestinal events that are most prominent at therapy initiation.^6^, ^9^, ^19^, ^31^ While the NICE guidance (NG28) and gastrointestinal tolerability are likely important drivers, early GLP‐1RA discontinuation is plausibly multifactorial and may also reflect perceived treatment effectiveness, complex dosing schedules, injection‐related barriers, limited access to diabetes education, economic constraints, and medication shortages.^19^, ^32^, ^33^, ^34^

The BMI‐stratified analyses indicate that, within the contemporary NICE framework (NG28), individuals with obesity, especially those with BMI ≥35 kg/m^2^, are more likely to persist with GLP‐1RA therapy and to reach higher doses. This suggests that dose escalation may be influenced by the therapeutic goals at the time of prescribing. When weight loss represents a primary treatment goal, early and observable benefits may reinforce both patient motivation and clinician confidence, thereby supporting continued treatment and dose escalation.

By contrast, we observed no meaningful differences in persistence or dosing patterns by CVD history, despite robust evidence for cardioprotective benefits of GLP‐1RAs in patients with established CVD.^7^, ^8^, ^9^, ^10^ Because cardiovascular benefits are not directly observable, whereas weight loss and adverse effects (e.g., gastrointestinal symptoms) are more tangible, both patients and clinicians may prioritize short‐term tolerability, contributing to early discontinuation.

Contemporary ADA‐EASD and European Society of Cardiology (ESC) guidelines strongly recommend early initiation of GLP‐1RAs or SGLT‐2is in patients with established or high risk of CVD.^4^, ^5^, ^35^ With 42% of patients with established CVD discontinuing GLP‐1RA therapy within 1‐year, we highlight a misalignment between cardiovascular prevention priorities and real‐world GLP‐1RA use in this high‐risk population. Consequently, our results emphasize the need for effective communication with patients regarding the importance of continued treatment and cardioprotection, together with proactive follow‐up to support persistence and appropriate dose escalation.

Finally, in addition to suboptimal persistence with treatment, we found that only 58% of semaglutide initiators had reached the recommended maintenance doses by the second prescription, while 21% remained on the starting dose, despite SPCs recommending escalation to maintenance doses after 1 month.^14^, ^15^ Escalation to higher semaglutide maintenance doses remained infrequent over time, with only 48.6% receiving 1.0 mg subcutaneous and 36.6% reaching 14 mg oral semaglutide at the tenth prescription, aligning with a previous cross‐sectional analysis by Barrett et al.^18^ using the UK‐based IQVIA Longitudinal Prescription Data (LRx).

Delay in dose escalation and continued use of lower doses across agents may reflect patient preferences, inter‐individual variation in treatment efficacy, concerns about tolerability (e.g., gastrointestinal events) or clinical inertia, defined as the failure of treatment intensification when clinically indicated.^36^ Additionally, the recent introduction of higher dulaglutide doses (in 2021) may explain their limited uptake.^37^

Leveraging a UK‐representative, longitudinal primary care database, we captured agent‐specific GLP‐1RA therapy utilization over time, allowing for clinically nuanced insights across relevant strata, including BMI, CVD history, and sex assigned at birth. By assessing longitudinal and dynamic patterns of discontinuation and dose titration at the prescription level, our analysis offers a more comprehensive understanding of treatment trajectories extending beyond the static insights from previous cross‐sectional studies.

However, important limitations must be considered. First, UK primary care databases such as the IMRD do not capture prescribing indication, treatment intent, or clinical priorities, nor do they include prescriptions issued in specialist care, where GLP‐1RAs may be initiated for obesity management.^38^ Consequently, we were not able to disentangle whether observed prescribing patterns represented, for instance, delayed dose escalation or deliberate, therapeutic target‐driven individualization of GLP‐1RA therapy (e.g., prioritizing weight loss, tolerability, or perceived benefit). In addition, because our analyses focused on prescribing patterns and their variation across key clinical subgroups, we did not evaluate the influence of clinical events, symptoms, or adverse effects occurring after treatment initiation on subsequent persistence or dose adjustment. Second, the IMRD provides information on prescriptions issued by GPs, but not whether medications were dispensed or taken. Third, we were unable to include more recently introduced therapies, such as tirzepatide or the 2.0 mg dose of semaglutide, which were not prescribed within the IMRD during the study period. Similarly, we could not assess dose trajectories for other agents such as liraglutide due to missing dose information. Finally, selection bias cannot be excluded in BMI‐subgroup analyses, as inclusion required the availability of a recent measurement.

Cumulatively, this study demonstrated that GLP‐1RA persistence in UK primary care was suboptimal, with 41% of initiators discontinuing within 1‐year; discontinuation remained similarly high among patients with established CVD (42%), while discontinuation was lower among individuals with obesity, particularly those with BMI ≥35 kg/m^2^ (37%). This finding stands in stark contrast with current ADA‐EASD and ESC guidelines, which advocate for GLP‐1RAs to reduce cardiovascular risk.^4^, ^5^, ^35^ The level of discontinuation among patients with established CVD reveals a substantial evidence‐practice gap in the management of this high‐risk group and highlights the absence of CVD‐specific GLP‐1RA recommendations in contemporary UK guidance (NG28).^6^ Closing this gap requires a paradigm shift in how these therapies are positioned to patients, emphasizing their role in disease modification and cardiovascular risk reduction alongside glycaemic control and weight management. Early follow‐up after initiation, coupled with clear communication on the long‐term cardiovascular benefits of GLP‐1RAs and proactive education on balancing benefits against side effects, could improve treatment persistence. Moreover, future research should explore patient‐ and provider‐level barriers to persistence and dose optimization, and assess the impact of individual dosing trajectories on clinical outcomes in routine care, including emerging agents such as high‐dose semaglutide and tirzepatide.

## AUTHOR CONTRIBUTIONS

AMB and FSU were responsible for study conception. AMB and FSU designed the study. FSU performed the statistical analyses. All authors contributed to the interpretation of the data. AMB and FSU wrote the manuscript. All authors contributed to important and critical edits of the manuscript draft and approved the final manuscript. AMB and FSU had full access to all the data in the study and take responsibility for the integrity of the data and the accuracy of the data analysis. The corresponding author attests that all listed authors meet authorship criteria and that no others meeting the criteria have been omitted. AMB and FSU are the guarantors of this study.

## CONFLICT OF INTEREST STATEMENT

The authors declare no conflicts of interest.

## ETHICS STATEMENT

This study was approved by the IQVIA Scientific Review Committee (23SRC017), and ethical approval for using the IQVIA Medical Research Data (IMRD) incorporating data from THIN, a Cegedim Database, was granted by the NHS Health Research Authority (East Midlands‐Derby Research Ethics Committee reference: 23/EM/0151).^20^

## Supporting information


**Data S1.** Supporting Information.

## Data Availability

The database, the IQVIA Medical Research Data (IMRD) incorporating data from THIN, a Cegedim Database, used in this study was accessed under license and cannot be publicly shared due to data protection and licensing restrictions. Researchers may apply for data access directly through IQVIA.
