# Supplementary material for: Real‐world persistence and dose titration of GLP‐1 receptor agonists in type 2 diabetes: A UK population‐based cohort study by obesity and cardiovascular disease status
Source: Diabetes Obes Metab. 2026 Feb 17;28(4):3386–95. doi: 10.1111/dom.70535 (PMC12992168; doi:10.1111/dom.70535)
Supplement: Supplementary file 1 — Data S1. Supporting Information. [file DOM-28-3386-s001.docx]

Online-Only Supplementary Material

Table of Contents

[SUPPLEMENTARY TABLES 3](#_Toc221280018)

[Table S1. ATC codes and generic names to define glucagon-like peptide-1 (GLP-1) receptor agonist use, as well as mode of administration and dose per administration to determine dosing. 3](#_Toc221280019)

[Table S2. Classes of glucose-lowering therapies defined by ATC codes and agents. 5](#_Toc221280020)

[Table S3. Defining baseline characteristics and variables in the multivariable cause-specific Cox model. 6](#_Toc221280021)

[Table S4. Characteristics of individuals initiating glucagon-like peptide-1 (GLP-1) receptor agonist therapy between 2018-2022, stratified by liraglutide, exenatide, and lixisenatide (extension of Table 1). 8](#_Toc221280022)

[Table S5. Characteristics of individuals initiating semaglutide between 2018-2022, overall and stratified by mode of administration (i.e., subcutaneous injection versus oral tablet). 9](#_Toc221280023)

[Table S6. Discontinuation of glucagon-like peptide-1 receptor agonist (GLP-1RA) therapy using different adherence definitions. 10](#_Toc221280024)

[Table S7. Discontinuation of glucagon-like peptide-1 receptor agonist (GLP-1RA) therapy by subgroups of cardiovascular disease (CVD) history, body-mass index (BMI) categories*, and sex assigned at birth. 11](#_Toc221280025)

[Table S8. Discontinuation of glucagon-like peptide-1 receptor agonist (GLP-1RA) therapy by subgroups of index GLP-1RA agent. 12](#_Toc221280026)

[Table S9. Dose titration of semaglutide from the first prescription (Rx) to the tenth prescription, overall and stratified by mode of administration (i.e., subcutaneous injection versus oral tablet). 13](#_Toc221280027)

[Table S10. Dose titration of semaglutide from the first prescription (Rx) to the tenth prescription, stratified by mode of administration (i.e., subcutaneous injection versus oral tablet) and body-mass index (BMI) categories*. 15](#_Toc221280028)

[Table S11. Dose titration of semaglutide from the first prescription (Rx) to the tenth prescription, stratified by mode of administration (i.e., subcutaneous injection versus oral tablet) and cardiovascular disease (CVD) history. 17](#_Toc221280029)

[Table S12. Dose titration of semaglutide from the first prescription (Rx) to the tenth prescription, stratified by mode of administration (i.e., subcutaneous injection versus oral tablet) and sex assigned at birth. 19](#_Toc221280030)

[Table S13. Dose titration of dulaglutide from the first prescription (Rx) to the tenth prescription, overall and stratified by body-mass index (BMI) categories* 21](#_Toc221280031)

[Table S14. Dose titration of dulaglutide from the first prescription (Rx) to the tenth prescription, overall and by subgroups of cardiovascular disease (CVD) history and sex assigned at birth. 22](#_Toc221280032)

[Table S15. Characteristics linked to glucagon-like peptide-1 (GLP-1) receptor agonist therapy discontinuation within 1-year. 24](#_Toc221280033)

[SUPPLEMENTARY FIGURES 25](#_Toc221280034)

[Figure S1. Cohort construction of individuals initiating glucagon-like peptide-1 (GLP-1) receptor agonists using the IQVIA Medical Research Data (IMRD) incorporating data from THIN, A Cegedim Database. 25](#_Toc221280035)

[SUPPLEMENTARY REFERENCES 26](#_Toc221280036)

# SUPPLEMENTARY TABLES

| Table S1. ATC codes and generic names to define glucagon-like peptide-1 (GLP-1) receptor agonist use, as well as mode of administration and dose per administration to determine dosing. | | | | |
| --- | --- | --- | --- | --- |
| **GLP-1 receptor agonist agent (ATC code)** | **Generic name** | **Combination product with insulin** | **Mode of administration: subcutaneous injection (sc) or oral tablet (po)** | **Dose per administration*** |
| **Semaglutide (A10BJ06)** | Rybelsus 3mg tablets | No | po | 3mg |
|  | Semaglutide 3mg tablets | No | po | 3mg |
|  | Rybelsus 7mg tablets | No | po | 7mg |
|  | Semaglutide 7mg tablets | No | po | 7mg |
|  | Rybelsus 14mg tablets | No | po | 14mg |
|  | Semaglutide 14mg tablets | No | po | 14mg |
|  | Ozempic 1mg/0.74ml solution for injection 3ml pre-filled pen | No | sc | 1mg |
|  | Semaglutide 1mg/0.74ml solution for injection 3ml pre-filled disposable device | No | sc | 1mg |
|  | Ozempic 0.5mg/0.37ml solution for injection 1.5ml pre-filled pen | No | sc | 0.5mg |
|  | Semaglutide 0.5mg/0.37ml solution for injection 1.5ml pre-filled disposable device | No | sc | 0.5mg |
|  | Ozempic 0.25mg/0.19ml solution for injection 1.5ml pre-filled pen | No | sc | 0.25mg |
|  | Semaglutide 0.25mg/0.19ml solution for injection 1.5ml pre-filled disposable device | No | sc | 0.25mg |
| **Dulaglutide (A10BJ05)** | Trulicity 3mg/0.5ml solution for injection pre-filled pens | No | sc | 3mg |
|  | Dulaglutide 3mg/0.5ml solution for injection pre-filled disposable devices | No | sc | 3mg |
|  | Trulicity 4.5mg/0.5ml solution for injection pre-filled pens | No | sc | 4.5mg |
|  | Dulaglutide 4.5mg/0.5ml solution for injection pre-filled disposable devices | No | sc | 4.5mg |
|  | Dulaglutide 1.5mg/0.5ml solution for injection pre-filled disposable devices | No | sc | 1.5mg |
|  | Dulaglutide 0.75mg/0.5ml solution for injection pre-filled disposable devices | No | sc | 0.5mg |
| **Liraglutide (A10BJ02)** | Liraglutide 6mg/ml solution for injection 3ml pre-filled disposable devices | No | sc | Pen provides different dosing options^1^ |
|  | Insulin degludec 100units/ml / Liraglutide 3.6mg/ml solution for injection 3ml pre-filled disposable devices | Yes | sc | Pen provides different dosing options^1^ |
| **Exenatide (A10BJ01)** | Bydureon BCise 2mg/0.85ml prolonged-release suspension for injection pre-filled pens | No | sc | 2mg |
|  | Exenatide 2mg/0.85ml prolonged-release suspension for injection pre-filled disposable devices | No | sc | 2mg |
|  | Exenatide 2mg powder and solvent for prolonged-release suspension for injection pre-filled disposable devices | No | sc | 2mg |
|  | Exenatide 2mg injection | No | sc | 2mg |
|  | Exenatide 2mg powder and solvent for prolonged-release suspension for injection vials | No | sc | 2mg |
|  | Exenatide 10micrograms/0.04ml solution for injection 2.4ml pre-filled disposable devices | No | sc | 10mcg |
|  | Exenatide 5micrograms/0.02ml solution for injection 1.2ml pre-filled disposable devices | No | sc | 5mcg |
| **Lixisenatide (A10BJ03)** | Lixisenatide 10micrograms/0.2ml solution for injection 3ml pre-filled disposable devices | No | sc | Discontinued on 26-May-2023^2^ |
|  | Lixisenatide 10micrograms/0.2ml solution for injection 3ml pre-filled disposable devices and Lixisenatide 20micrograms/0 | No | sc | Discontinued on 26-May-2023^2^ |
|  | Lixisenatide 20micrograms/0.2ml solution for injection 3ml pre-filled disposable devices | No | sc | Discontinued on 26-May-2023^2^ |
|  | Insulin glargine 100units/ml / Lixisenatide 33micrograms/ml solution for injection 3ml pre-filled disposable devices | Yes | sc | Different dosing options^3^ |
|  | Suliqua 100units/ml / 50micrograms/ml solution for injection 3ml pre-filled SoloStar pen | Yes | sc | Different dosing options^3^ |
|  | Suliqua 100units/ml / 33micrograms/ml solution for injection 3ml pre-filled SoloStar pen | Yes | sc | Different dosing options^3^ |
| **Albiglutide (A10BJ04)** | Albiglutide 30mg powder and solvent for solution for injection pre-filled disposable devices | No | sc | Discontinued on 27-Jun-2018^4^ |
| *Dose per administration is based on the information provided by the generic name. Frequency of administration is assumed to align with the corresponding Summary of Product Characteristics (SPC). For subcutaneous (sc) formulations, if a pre-filled pen device allows for multiple dosing options or if the product has been discontinued, the dose per administration is considered unknown. | | | | |

| Table S2. Classes of glucose-lowering therapies defined by ATC codes and agents. | | | |
| --- | --- | --- | --- |
| **Drug class** | **Drug class ATC code** | **Agent** | **Agent ATC code** |
| Biguanides | A10BA | Metformin | A10BA02 |
| Sulfonylureas | A10BB | Glibenclamide | A10BB01 |
|  |  | Chlorpropamide | A10BB02 |
|  |  | Tolbutamide | A10BB03 |
|  |  | Tolazamide | A10BB05 |
|  |  | Gliclazide | A10BB09 |
|  |  | Glimepiride | A10BB12 |
| Thiazolidinediones | A10BG | Troglitazone | A10BG01 |
|  |  | Rosiglitazone | A10BG02 |
|  |  | Pioglitazone | A10BG03 |
| Dipeptidyl peptidase 4 (DPP-4) inhibitors | A10BH | Sitagliptin | A10BH01 |
|  |  | Vildagliptin | A10BH02 |
|  |  | Saxagliptin | A10BH03 |
|  |  | Alogliptin | A10BH04 |
|  |  | Linagliptin | A10BH05 |
| Glucagon-like peptide-1 (GLP-1) receptor agonists | A10BJ | Exenatide | A10BJ01 |
|  |  | Liraglutide | A10BJ02 |
|  |  | Lixisenatide | A10BJ03 |
|  |  | Albiglutide | A10BJ04 |
|  |  | Dulaglutide | A10BJ05 |
|  |  | Semaglutide | A10BJ06 |
| Sodium-glucose co-transporter 2 (SGLT-2) inhibitors | A10BK | Dapagliflozin | A10BK01 |
|  |  | Canagliflozin | A10BK02 |
|  |  | Empagliflozin | A10BK03 |
|  |  | Ertugliflozin | A10BK04 |
| Insulin | A10A | All agents identified by an ATC code starting with “A10A” | ATC codes starting with “A10A” |
| Other glucose-lowering drugs | Not applicable | Acarbose | A10BF01 |
|  |  | Repaglinide | A10BX02 |
|  |  | Nateglinide | A10BX03 |
|  |  | Guar gum | A10BX01 |

| Table S3. Defining baseline characteristics and variables in the multivariable cause-specific Cox model. | | |
| --- | --- | --- |
| **Characteristics** | **Included in baseline characteristics** | **Included in multivariable cause-specific Cox model** |
| Age at initiation, years, median (IQR) | Yes | No |
| Age <30, years, n (%) | No | Yes |
| Age 30-<50, years, n (%) | No | Yes |
| Age 50-<60, years, n (%) | No | Yes |
| Age 60-<70, years, n (%) | No | Yes |
| ≥70, years, n (%) | No | Yes |
| Female sex assigned at birth, n (%) | Yes | Yes |
| Type 2 diabetes duration^a^, years, median (IQR) | Yes | Dichotomized based on median |
| **Previous use of glucose-lowering therapies^b^, n (%)** | | |
| Metformin | Yes | No |
| Sulfonylureas | Yes | No |
| SGLT-2 inhibitors | Yes | No |
| DPP-4 inhibitors | Yes | No |
| Thiazolidinediones | Yes | No |
| Insulin | Yes | No |
| Other GLT | Yes | No |
| ≥3 GLT | Yes | No |
| **Concurrent use of glucose-lowering therapies^c^, n (%)** | | |
| Metformin | Yes | Yes |
| Sulfonylureas | Yes | Yes |
| SGLT-2 inhibitors | Yes | Yes |
| DPP-4 inhibitors | Yes | Yes |
| Thiazolidinediones | Yes | Yes |
| Insulin | Yes | Yes |
| Other GLT | Yes | Yes |
| ≥3 GLT | Yes | Yes |
| **Comorbidities^d^, n (%)** | | |
| Cardiovascular disease | Yes | Yes |
| Myocardial infarction | Yes | No |
| Stroke | Yes | No |
| Heart failure | Yes | No |
| Hypertension | Yes | Yes |
| Dyslipidemia | Yes | Yes |
| Sleep apnea | Yes | No |
| Asthma | Yes | Yes |
| Chronic obstructive pulmonary disease | Yes | Yes |
| Chronic kidney disease | Yes | Yes |
| Osteoarthritis | Yes | No |
| Depression | Yes | Yes |
| Cancer | Yes | No |
| **Comedications^e^, n (%)** | | |
| Statins | Yes | No |
| Angiotensin converting enzyme inhibitors | Yes | No |
| Angiotensin II receptor blockers | Yes | No |
| Diuretics | Yes | No |
| Calcium channel blockers | Yes | No |
| Beta-blockers | Yes | No |
| Acetylsalicylic acid | Yes | No |
| Other antiplatelets | Yes | No |
| Anticoagulants | Yes | No |
| **Laboratory and vital sign measurements^f^** | | |
| BMI, kg/m^2^, median (IQR) | Yes | No |
| BMI <30 kg/m^2^, n (%) | Yes | Yes |
| BMI 30-<35 kg/m^2^, n (%) | Yes | Yes |
| BMI ≥35 kg/m^2^, n (%) | Yes | Yes |
| BMI missing, n (%) | Yes | No |
| eGFR, ml/min per 1.72m^2^, median (IQR) | Yes | Dichotomized based on median |
| eGFR missing, n (%) | Yes | Yes |
| HbA1c, %, median (IQR) | Yes | Dichotomized based on median |
| HbA1c missing, n (%) | Yes | Yes |
| Systolic blood pressure, median (IQR), mmHg | Yes | No |
| Diastolic blood pressure, median (IQR), mmHg | Yes | No |
| Serum cholesterol, median (IQR), mmol/L | Yes | No |
| **Lifestyle factors^g^, n (%)** | | |
| Markers of current smoking | Yes | No |
| Markers of current alcohol use | Yes | No |
| Abbreviations: SGLT-2 sodium-glucose co-transporter 2, DPP-4 dipeptidyl peptidase-4, HbA1c glycated hemoglobin, BMI body-mass index, eGFR estimated glomerular filtration rate. ^a^Time (years) between the first non-insulin glucose-lowering therapy initiated and GLP-1 receptor agonist initiation. ^b^Defined as having at least one prescription for a glucose-lowering therapy specified in Supplementary Table S2 before GLP-1 receptor agonist initiation. ^c^Defined as having at least one prescription for a glucose-lowering therapy specified in Supplementary Table S2 within the 180 days before or at GLP-1 receptor agonist initiation. ^d^Diagnosis recorded before or at GLP-1 receptor agonist initiation. ^e^Defined as having at least one prescription within the 365 days before or at GLP-1 receptor agonist initiation. ^f^Most recent measurement recorded within the 365 days before or at GLP-1 receptor agonist initiation, with proportions of missing values reported. The eGFR was calculated according to the 2021 Chronic Kidney Disease Epidemiology Collaboration (CKD-EPI) equation based on serum creatinine.^5^ ^g^Most recent record before or at GLP-1 receptor agonist initiation. | | |

| Table S4. Characteristics of individuals initiating glucagon-like peptide-1 (GLP-1) receptor agonist therapy between 2018-2022, stratified by liraglutide, exenatide, and lixisenatide (extension of Table 1). | | | |
| --- | --- | --- | --- |
| **Characteristics** | **Liraglutide** | **Exenatide** | **Lixisenatide** |
| Individuals included, n (%) | 974 (11.9) | 206 (2.5) | 35 (0.4) |
| Age at initiation, years, median (IQR) | 59.9 [52.5, 66.7] | 59.9 [53.7, 67.0] | 59.1 [50.7, 63.7] |
| Female sex assigned at birth, n (%) | 475 (48.8) | 91 (44.2) | 19 (54.3) |
| Type 2 diabetes duration, years, median (IQR) | 7.2 [3.6, 11.9] | 6.3 [3.0, 10.8] | 6.2 [4.0, 8.8] |
| **Previous use of GLT*, n (%)** | | | |
| Metformin | 944 (96.9) | 204 (99.0) | 35 (100.0) |
| Sulfonylureas | 665 (68.3) | 112 (54.4) | 24 (68.6) |
| SGLT-2 inhibitors | 492 (50.5) | 113 (54.9) | 17 (48.6) |
| DPP-4 inhibitors | 620 (63.7) | 105 (51.0) | 22 (62.9) |
| Thiazolidinediones | 200 (20.5) | 35 (17.0) | <7 |
| Insulin | 173 (17.8) | 27 (13.1) | <7 |
| Other GLT | 19 (2.0) | <7 | <7 |
| ≥3 GLT | 724 (74.3) | 129 (62.6) | 29 (82.9) |
| **Concurrent use of GLT†, n (%)** | | | |
| Metformin | 799 (82.0) | 181 (87.9) | 28 (80.0) |
| Sulfonylureas | 478 (49.1) | 78 (37.9) | 21 (60.0) |
| SGLT-2 inhibitors | 396 (40.7) | 86 (41.7) | 14 (40.0) |
| DPP-4 inhibitors | 463 (47.5) | 77 (37.4) | 17 (48.6) |
| Thiazolidinediones | 51 (5.2) | 10 (4.9) | <7 |
| Insulin | 162 (16.6) | 20 (9.7) | <7 |
| Other GLT | <7 | <7 | <7 |
| ≥3 GLT | 485 (49.8) | 86 (41.7) | 14 (40.0) |
| **Comorbidities‡, n (%)** | | | |
| Cardiovascular disease | 212 (21.8) | 41 (19.9) | <7 |
| Hypertension | 627 (64.4) | 135 (65.5) | 22 (62.9) |
| Dyslipidemia | 189 (19.4) | 32 (15.5) | <7 |
| Depression | 417 (42.8) | 82 (39.8) | 16 (45.7) |
| Asthma | 200 (20.5) | 31 (15.0) | 12 (34.3) |
| Chronic obstructive pulmonary disease | 251 (25.8) | 36 (17.5) | 12 (34.3) |
| Chronic kidney disease | 192 (19.7) | 22 (10.7) | <7 |
| **Laboratory and vital sign measurements§** | | | |
| BMI, kg/m^2^, median (IQR) | 34.9 [31.4, 38.7] | 35.0 [30.8, 38.3] | 32.7 [31.2, 36.0] |
| BMI 30-<35 kg/m^2^, n (%) | 259 (26.6) | 56 (27.2) | 13 (37.1) |
| BMI ≥35 kg/m^2^, n (%) | 358 (36.8) | 87 (42.2) | 9 (25.7) |
| BMI missing, n (%) | 238 (24.4) | 33 (16.0) | 7 (20.0) |
| eGFR, ml/min per 1.72m^2^, median (IQR) | 99.1 [78.4, 109.6] | 98.5 [87.2, 108.0] | 103.0 [78.4, 114.9] |
| eGFR<60 ml/min per 1.72m^2^, n (%) | 106 (10.9) | 14 (6.8) | <7 |
| eGFR missing, n (%) | 37 (3.8) | 13 (6.3) | <7 |
| HbA1c, %, median (IQR) | 8.2 [7.6, 8.6] | 8.2 [7.7, 8.6] | 8.4 [8.0, 8.7] |
| HbA1c missing, n (%) | 391 (40.1) | 85 (41.3) | 10 (28.6) |
| Abbreviations: GLT glucose-lowering therapies, SGLT-2 sodium-glucose co-transporter 2, DPP-4 dipeptidyl peptidase-4, HbA1c glycated hemoglobin, BMI body-mass index, eGFR estimated glomerular filtration rate.  *Defined as having at least one prescription for a glucose-lowering therapy specified in Supplementary Table S2 before GLP-1 receptor agonist initiation. †Defined as having at least one prescription for a glucose-lowering therapy specified in Supplementary Table S2 within the 180 days before or at GLP-1 receptor agonist initiation. ‡Diagnosis recorded before or at GLP-1 receptor agonist initiation. §Most recent measurement recorded within the year before or at GLP-1 receptor agonist initiation. | | | |

| Table S5. Characteristics of individuals initiating semaglutide between 2018-2022, overall and stratified by mode of administration (i.e., subcutaneous injection versus oral tablet). | | | |
| --- | --- | --- | --- |
| **Characteristics** | **Semaglutide** | **Subcutaneous injection (sc)** | **Oral Tablet (po)** |
| Individuals included, n (%) | 3173 (100.0) | 2417 (76.2) | 756 (23.8) |
| Age at initiation, years, median (IQR) | 60.6 [52.7, 68.7] | 59.8 [52.0, 68.1] | 63.4 [55.8, 71.5] |
| Female sex assigned at birth, n (%) | 1381 (43.5) | 1091 (45.1) | 290 (38.4) |
| Type 2 diabetes duration, years, median (IQR) | 6.8 [3.3, 11.5] | 6.6 [3.1, 11.3] | 7.5 [3.8, 12.2] |
| **Previous use of GLT*, n (%)** | | | |
| Metformin | 3087 (97.3) | 2353 (97.4) | 734 (97.1) |
| Sulfonylureas | 1712 (54.0) | 1279 (52.9) | 433 (57.3) |
| SGLT-2 inhibitors | 1895 (59.7) | 1390 (57.5) | 505 (66.8) |
| DPP-4 inhibitors | 1874 (59.1) | 1402 (58.0) | 472 (62.4) |
| Thiazolidinediones | 495 (15.6) | 362 (15.0) | 133 (17.6) |
| Insulin | 402 (12.7) | 348 (14.4) | 54 (7.1) |
| Other GLT | 38 (1.2) | 30 (1.2) | 8 (1.1) |
| ≥3 GLT | 2118 (66.8) | 1580 (65.4) | 538 (71.2) |
| **Concurrent use of GLT†, n (%)** | | | |
| Metformin | 2721 (85.8) | 2085 (86.3) | 636 (84.1) |
| Sulfonylureas | 1176 (37.1) | 899 (37.2) | 277 (36.6) |
| SGLT-2 inhibitors | 1563 (49.3) | 1137 (47.0) | 426 (56.3) |
| DPP-4 inhibitors | 1450 (45.7) | 1074 (44.4) | 376 (49.7) |
| Thiazolidinediones | 142 (4.5) | 104 (4.3) | 38 (5.0) |
| Insulin | 353 (11.1) | 311 (12.9) | 42 (5.6) |
| Other GLT | <7 | <7 | <7 |
| ≥3 GLT | 1430 (45.1) | 1078 (44.6) | 352 (46.6) |
| **Comorbidities‡, n (%)** | | | |
| Cardiovascular disease | 569 (17.9) | 424 (17.5) | 145 (19.2) |
| Hypertension | 2027 (63.9) | 1556 (64.4) | 471 (62.3) |
| Dyslipidemia | 622 (19.6) | 475 (19.7) | 147 (19.4) |
| Depression | 1287 (40.6) | 1016 (42.0) | 271 (35.8) |
| Asthma | 727 (22.9) | 556 (23.0) | 171 (22.6) |
| Chronic obstructive pulmonary disease | 819 (25.8) | 629 (26.0) | 190 (25.1) |
| Chronic kidney disease | 511 (16.1) | 384 (15.9) | 127 (16.8) |
| **Laboratory and vital sign measurements§** | | | |
| BMI, kg/m^2^, median (IQR) | 34.0 [30.5, 38.1] | 34.5 [31.0, 38.52] | 32.2 [28.9, 36.4] |
| BMI 30-<35 kg/m^2^, n (%) | 847 (26.7) | 644 (26.6) | 203 (26.9) |
| BMI ≥35 kg/m^2^, n (%) | 1027 (32.4) | 849 (35.1) | 178 (23.5) |
| BMI missing, n (%) | 791 (24.9) | 597 (24.7) | 194 (25.7) |
| eGFR, ml/min per 1.72m^2^, median (IQR) | 98.2 [80.2, 107.6] | 98.9 [81.5, 108.6] | 95.7 [78.4, 104.7] |
| eGFR<60 ml/min per 1.72m^2^, n (%) | 333 (10.5) | 240 (9.9) | 93 (12.3) |
| eGFR missing, n (%) | 122 (3.8) | 87 (3.6) | 35 (4.6) |
| HbA1c, %, median (IQR) | 8.1 [7.6, 8.6] | 8.1 [7.5, 8.6] | 8.2 [7.7, 8.6] |
| HbA1c missing, n (%) | 1231 (38.8) | 914 (37.8) | 317 (41.9) |
| Abbreviations: GLT glucose-lowering therapies, SGLT-2 sodium-glucose co-transporter 2, DPP-4 dipeptidyl peptidase-4, HbA1c glycated hemoglobin, BMI body-mass index, eGFR estimated glomerular filtration rate.  *Defined as having at least one prescription for a glucose-lowering therapy specified in Supplementary Table S2 before GLP-1 receptor agonist initiation. †Defined as having at least one prescription for a glucose-lowering therapy specified in Supplementary Table S2 within the 180 days before or at GLP-1 receptor agonist initiation. ‡Diagnosis recorded before or at GLP-1 receptor agonist initiation. §Most recent measurement recorded within the year before or at GLP-1 receptor agonist initiation. | | | |

| Table S6. Discontinuation of glucagon-like peptide-1 receptor agonist (GLP-1RA) therapy using different adherence definitions. | | | | | | |
| --- | --- | --- | --- | --- | --- | --- |
| **Time from GLP-1RA initiation, years** | **GLP-1RA therapy discontinued (i.e., stopped* / interrupted†)** | **GLP-1RA therapy stopped*** | **GLP-1RA therapy interrupted†** | **Death** | **Censored or GLP-1RA therapy discontinued** | **Absolute risk of discontinuation, % (95% CI)** |
| **Treatment gap: 60-days**† | | | | | | |
| 0.5 | 3378 (41.2) | 1282 (15.6) | 2096 (25.6) | 14 (0.2) | 3467 (42.3) | 41.2 (40.1-42.3) |
| 1 | 4679 (57.1) | 1730 (21.1) | 2949 (36.0) | 28 (0.3) | 4821 (58.8) | 57.0 (55.9-58.0) |
| **Treatment gap: 90-days**† | | | | | | |
| 0.5 | 2291 (27.9) | 1355 (16.5) | 936 (11.4) | 15 (0.2) | 2385 (29.1) | 27.9 (27.0-28.9) |
| 1 | 3382 (41.2) | 1972 (24.0) | 1410 (17.2) | 34 (0.4) | 3544 (43.2) | 41.1 (40.1-42.2) |
| **Treatment gap: 120-days**† | | | | | | |
| 0.5 | 1949 (23.8) | 1375 (16.8) | 574 (7.0) | 15 (0.2) | 2043 (24.9) | 23.8 (22.8-24.7) |
| 1 | 2901 (35.4) | 2046 (25.0) | 855 (10.4) | 36 (0.4) | 3070 (37.4) | 35.3 (34.2-36.3) |
| *Individuals were classified as having stopped GLP-1RA therapy if there were no further GLP-1RA prescriptions. †Individuals were classified as having interrupted GLP-1RA therapy if the gap between consecutive GLP-1RA prescriptions exceeded the interval specified in respective definitions. | | | | | | |

| Table S7. Discontinuation of glucagon-like peptide-1 receptor agonist (GLP-1RA) therapy by subgroups of cardiovascular disease (CVD) history, body-mass index (BMI) categories*, and sex assigned at birth. | | | | | | |
| --- | --- | --- | --- | --- | --- | --- |
| **Time from GLP-1RA initiation, years** | **GLP-1RA therapy discontinued (i.e., stopped† / interrupted‡)** | **GLP-1RA therapy stopped†** | **GLP-1RA therapy interrupted‡** | **Death** | **Censored or GLP-1RA therapy discontinued** | **Absolute risk of discontinuation, % (95% CI)** |
| **CVD history (n=1565), n (%)** | | | | | | |
| 0.5 | 465 (29.7) | 298 (19.0) | 167 (10.7) | <7 | 487 (31.1) | 29.5 (27.3-31.8) |
| 1 | 661 (42.2) | 424 (27.1) | 237 (15.1) | 12 (0.8) | 693 (44.3) | 42.1 (39.7-44.6) |
| **No CVD history (n=7735), n (%)** | | | | | | |
| 0.5 | 1826 (27.5) | 1057 (15.9) | 769 (11.6) | 9 (0.1) | 1898 (28.6) | 27.5 (26.4-28.6) |
| 1 | 2721 (41.0) | 1548 (23.3) | 1173 (17.7) | 22 (0.3) | 2851 (43.0) | 40.9 (39.7-42.1) |
| **BMI ≥35 kg/m^2^ (n=2712), n (%)** | | | | | | |
| 0.5 | 673 (24.8) | 380 (14.0) | 293 (10.8) | <7 | 706 (26.0) | 24.8 (23.2-26.4) |
| 1 | 1010 (37.2) | 547 (20.2) | 463 (17.1) | 10 (0.4) | 1065 (39.3) | 37.2 (35.4-39.1) |
| **BMI 30-<35 kg/m^2^ (n=2251), n (%)** | | | | | | |
| 0.5 | 622 (27.6) | 370 (16.4) | 252 (11.2) | <7 | 643 (28.6) | 27.6 (25.8-29.5) |
| 1 | 912 (40.5) | 556 (24.7) | 356 (15.8) | 8 (0.4) | 953 (42.3) | 40.4 (38.4-42.4) |
| **BMI <30 kg/m^2^ (n=1282), n (%)** | | | | | | |
| 0.5 | 438 (34.2) | 289 (22.5) | 149 (11.6) | <7 | 452 (35.3) | 34.2 (31.6-36.8) |
| 1 | 634 (49.5) | 408 (31.8) | 226 (17.6) | <7 | 659 (51.4) | 49.2 (46.5-52.0) |
| **Female (n=3695), n (%)** | | | | | | |
| 0.5 | 1037 (28.1) | 625 (16.9) | 412 (11.2) | <7 | 1073 (29.0) | 28.1 (26.6-29.5) |
| 1 | 1523 (41.2) | 892 (24.1) | 631 (17.1) | 14 (0.4) | 1591 (43.1) | 41.2 (39.6-42.8) |
| **Male (n=4505), n (%)** | | | | | | |
| 0.5 | 1254 (27.8) | 730 (16.2) | 524 (11.6) | 10 (0.2) | 1312 (29.1) | 27.8 (26.5-29.1) |
| 1 | 1859 (41.3) | 1080 (24.0) | 779 (17.3) | 20 (0.4) | 1953 (43.4) | 41.1 (39.7-42.5) |
| *Most recent measurement recorded within the year before or at GLP-1RA initiation. †Individuals were classified as having stopped GLP-1RA therapy if there were no further GLP-1RA prescriptions, while assuming a 30-day supply per prescription. ‡Individuals were classified as having interrupted GLP-1RA therapy if the gap between consecutive GLP-1RA prescriptions exceeded 90-days. | | | | | | |

| Table S8. Discontinuation of glucagon-like peptide-1 receptor agonist (GLP-1RA) therapy by subgroups of index GLP-1RA agent. | | | | | | |
| --- | --- | --- | --- | --- | --- | --- |
| **Time from GLP-1RA initiation, years** | **GLP-1RA therapy discontinued (i.e., stopped* / interrupted† / switched‡)** | **GLP-1RA therapy stopped*** | **GLP-1RA therapy interrupted†** | **GLP-1RA agent switched‡** | **Censored or GLP-1RA therapy discontinued** | **Absolute risk of discontinuation, % (95% CI)** |
| **GLP-1RA overall (n=8200), n (%)** | | | | | | |
| 0.5 | 2455 (29.9) | 1317 (16.1) | 921 (11.2) | 217 (2.6) | 2547 (31.1) | 29.9 (28.9-30.9) |
| 1 | 3611 (44.0) | 1899 (23.2) | 1374 (16.8) | 338 (4.1) | 3764 (45.9) | 43.9 (42.9-45.0) |
| **Semaglutide overall (n=3173), n (%)** | | | | | | |
| 0.5 | 1076 (33.9) | 587 (18.5) | 423 (13.3) | 66 (2.1) | 1111 (33.9) | 33.9 (32.3-35.6) |
| 1 | 1547 (48.8) | 854 (26.9) | 608 (19.2) | 85 (2.7) | 1601 (48.8) | 48.7 (46.9-50.4) |
| **Semaglutide subcutaneous injection (n=2417), n (%)** | | | | | | |
| 0.5 | 783 (32.4) | 430 (17.8) | 304 (12.6) | 49 (2.0) | 810 (33.5) | 32.4 (30.5-34.3) |
| 1 | 1125 (46.5) | 633 (26.2) | 428 (17.7) | 64 (2.6) | 1166 (48.2) | 46.4 (44.4-48.4) |
| **Semaglutide oral tablet (n=756), n (%)** | | | | | | |
| 0.5 | 293 (38.8) | 157 (20.8) | 119 (15.7) | 17 (2.2) | 301 (39.8) | 38.5 (35.0-42.0) |
| 1 | 422 (55.8) | 221 (29.2) | 180 (23.8) | 21 (2.8) | 435 (57.5) | 55.8 (52.3-59.4) |
| **Dulaglutide (n=3812), n (%)** | | | | | | |
| 0.5 | 923 (24.2) | 542 (14.2) | 304 (8.0) | 77 (2.0) | 968 (25.4) | 24.2 (22.9-25.6) |
| 1 | 1409 (37.0) | 809 (21.2) | 472 (12.4) | 128 (3.4) | 1485 (39.0) | 36.9 (35.4-38.4) |
| **Liraglutide (n=974), n (%)** | | | | | | |
| 0.5 | 362 (37.2) | 146 (15.0) | 170 (17.5) | 46 (4.7) | 372 (38.2) | 37.2 (34.1-40.2) |
| 1 | 527 (54.1) | 181 (18.6) | 254 (26.1) | 92 (9.4) | 546 (56.1) | 53.8 (50.7-56.9) |
| **Exenatide (n=206), n (%)** | | | | | | |
| 0.5 | 72 (35.0) | 31 (15.0) | 20 (9.7) | 21 (10.2) | 73 (35.4) | 35.0 (28.4-41.5) |
| 1 | 101 (49.0) | 41 (19.9) | 35 (17.0) | 25 (12.1) | 104 (50.5) | 48.5 (41.7-55.4) |
| **Lixisenatide (n=35), n (%)** | | | | | | |
| 0.5 | 22 (62.9) | 11 (31.4) | <7 | 7 (20.0) | 23 (65.7) | 62.9 (46.4-79.3) |
| 1 | 27 (77.1) | 14 (40.0) | <7 | 8 (22.9) | 28 (80.0) | 74.3 (59.3-89.3) |
| *Individuals were classified as having stopped therapy if there were no further prescriptions for the respective GLP-1RA agent. †Individuals were classified as having interrupted therapy if the gap between consecutive prescriptions exceeded 90-days. **‡**Individuals were classified as having switched therapy if they switched to a different GLP-1RA agent. | | | | | | |

| Table S9. Dose titration of semaglutide from the first prescription (Rx) to the tenth prescription, overall and stratified by mode of administration (i.e., subcutaneous injection versus oral tablet). | | | | |
| --- | --- | --- | --- | --- |
| **Semaglutide doses per prescription** | | **Semaglutide overall** | **Subcutaneous injection** | **Oral tablet** |
| **1^st^ prescription, n (%)** | | 3173 (100.0) | 2417 (100.0) | 756 (100.0) |
| Time since Rx 1, weeks, median [IQR] | | 0.00 [0.00, 0.00] | 0.00 [0.00, 0.00] | 0.00 [0.00, 0.00] |
| Oral tablet | 3 mg, n (%) | 655 (20.6) | <7 | 655 (86.6) |
|  | 7 mg, n (%) | 76 (2.4) | <7 | 76 (10.1) |
|  | 14 mg, n (%) | 25 (0.8) | <7 | 25 (3.3) |
| Subcutaneous injection | 0.25 mg, n (%) | 1997 (62.9) | 1997 (82.6) | <7 |
|  | 0.50 mg, n (%) | 345 (10.9) | 345 (14.3) | <7 |
|  | 1.00 mg, n (%) | 75 (2.4) | 75 (3.1) | <7 |
| **2^nd^ prescription, n (%)** | | 2792 (88.0) | 2153 (89.1) | 639 (84.5) |
| Time since Rx 1, weeks, median [IQR] | | 4.00 [3.14, 5.00] | 4.00 [3.14, 5.00] | 4.00 [3.14, 4.86] |
| Oral tablet | 3 mg, n (%) | 167 (5.3) | <7 | 162 (21.4) |
|  | 7 mg, n (%) | 440 (13.9) | <7 | 440 (58.2) |
|  | 14 mg, n (%) | 34 (1.1) | <7 | 34 (4.5) |
| Subcutaneous injection | 0.25 mg, n (%) | 531 (16.7) | 530 (21.9) | <7 |
|  | 0.50 mg, n (%) | 1422 (44.8) | 1420 (58.8) | <7 |
|  | 1.00 mg, n (%) | 198 (6.2) | 198 (8.2) | <7 |
| **3^rd^ prescription, n (%)** | | 2560 (80.7) | 1988 (82.3) | 572 (75.7) |
| Time since Rx 1, weeks, median [IQR] | | 8.00 [7.00, 9.86] | 8.00 [6.86, 9.86] | 8.29 [7.00, 10.57] |
| Oral tablet | 3 mg, n (%) | 109 (3.4) | <7 | 108 (14.3) |
|  | 7 mg, n (%) | 371 (11.7) | <7 | 368 (48.7) |
|  | 14 mg, n (%) | 90 (2.8) | <7 | 90 (11.9) |
| Subcutaneous injection | 0.25 mg, n (%) | 299 (9.4) | 296 (12.2) | <7 |
|  | 0.50 mg, n (%) | 1151 (36.3) | 1150 (47.6) | <7 |
|  | 1.00 mg, n (%) | 540 (17.0) | 538 (22.3) | <7 |
| **4^th^ prescription, n (%)** | | 2353 (74.2) | 1847 (76.4) | 506 (66.9) |
| Time since Rx 1, weeks, median [IQR] | | 12.14 [10.71, 14.86] | 12.00 [10.57, 14.14] | 12.86 [11.04, 16.57] |
| Oral tablet | 3 mg, n (%) | 78 (2.5) | <7 | 76 (10.1) |
|  | 7 mg, n (%) | 325 (10.2) | <7 | 322 (42.6) |
|  | 14 mg, n (%) | 101 (3.2) | <7 | 100 (13.2) |
| Subcutaneous injection | 0.25 mg, n (%) | 221 (7.0) | 218 (9.0) | <7 |
|  | 0.50 mg, n (%) | 952 (30.0) | 950 (39.3) | <7 |
|  | 1.00 mg, n (%) | 676 (21.3) | 673 (27.8) | <7 |
| **5^th^ prescription, n (%)** | | 2194 (69.1) | 1724 (71.3) | 470 (62.2) |
| Time since Rx 1, weeks, median [IQR] | | 16.29 [14.71, 19.71] | 16.14 [14.57, 19.00] | 17.36 [15.14, 22.79] |
| Oral tablet | 3 mg, n (%) | 62 (2.0) | <7 | 60 (7.9) |
|  | 7 mg, n (%) | 292 (9.2) | <7 | 289 (38.2) |
|  | 14 mg, n (%) | 113 (3.6) | <7 | 111 (14.7) |
| Subcutaneous injection | 0.25 mg, n (%) | 166 (5.2) | 161 (6.7) | <7 |
|  | 0.50 mg, n (%) | 859 (27.1) | 857 (35.5) | <7 |
|  | 1.00 mg, n (%) | 702 (22.1) | 699 (28.9) | <7 |
| **6^th^ prescription, n (%)** | | 2073 (65.3) | 1635 (67.6) | 438 (57.9) |
| Time since Rx 1, weeks, median [IQR] | | 20.43 [18.43, 24.43] | 20.14 [18.29, 23.57] | 22.07 [18.89, 29.00] |
| Oral tablet | 3 mg, n (%) | 55 (1.7) | <7 | 53 (7.0) |
|  | 7 mg, n (%) | 264 (8.3) | <7 | 259 (34.3) |
|  | 14 mg, n (%) | 114 (3.6) | <7 | 113 (14.9) |
| Subcutaneous injection | 0.25 mg, n (%) | 138 (4.3) | 134 (5.5) | <7 |
|  | 0.50 mg, n (%) | 797 (25.1) | 791 (32.7) | <7 |
|  | 1.00 mg, n (%) | 705 (22.2) | 702 (29.0) | <7 |
| **7^th^ prescription, n (%)** | | 1962 (61.8) | 1555 (64.3) | 407 (53.8) |
| Time since Rx 1, weeks, median [IQR] | | 24.57 [22.29, 29.00] | 24.29 [22.14, 27.86] | 26.71 [23.14, 35.07] |
| Oral tablet | 3 mg, n (%) | 41 (1.3) | <7 | 41 (5.4) |
|  | 7 mg, n (%) | 237 (7.5) | <7 | 234 (31.0) |
|  | 14 mg, n (%) | 121 (3.8) | <7 | 118 (15.6) |
| Subcutaneous injection | 0.25 mg, n (%) | 129 (4.1) | 128 (5.3) | <7 |
|  | 0.50 mg, n (%) | 732 (23.1) | 725 (30.0) | 7 (0.9) |
|  | 1.00 mg, n (%) | 702 (22.1) | 696 (28.8) | <7 |
| **8^th^ prescription, n (%)** | | 1859 (58.6) | 1483 (61.4) | 376 (49.7) |
| Time since Rx 1, weeks, median [IQR] | | 28.71 [26.29, 33.29] | 28.29 [26.00, 32.29] | 30.86 [27.14, 41.00] |
| Oral tablet | 3 mg, n (%) | 37 (1.2) | <7 | 37 (4.9) |
|  | 7 mg, n (%) | 201 (6.3) | <7 | 200 (26.5) |
|  | 14 mg, n (%) | 127 (4.0) | <7 | 124 (16.4) |
| Subcutaneous injection | 0.25 mg, n (%) | 108 (3.4) | 108 (4.5) | <7 |
|  | 0.50 mg, n (%) | 686 (21.6) | 677 (28.0) | 9 (1.2) |
|  | 1.00 mg, n (%) | 700 (22.1) | 694 (28.7) | <7 |
| **9^th^ prescription, n (%)** | | 1771 (55.8) | 1421 (58.8) | 350 (46.3) |
| Time since Rx 1, weeks, median [IQR] | | 32.86 [30.29, 37.71] | 32.43 [30.14, 36.71] | 34.86 [31.14, 46.86] |
| Oral tablet | 3 mg, n (%) | 33 (1.0) | <7 | 33 (4.4) |
|  | 7 mg, n (%) | 179 (5.6) | <7 | 177 (23.4) |
|  | 14 mg, n (%) | 125 (3.9) | <7 | 123 (16.3) |
| Subcutaneous injection | 0.25 mg, n (%) | 99 (3.1) | 98 (4.1) | <7 |
|  | 0.50 mg, n (%) | 641 (20.2) | 632 (26.1) | 9 (1.2) |
|  | 1.00 mg, n (%) | 694 (21.9) | 687 (28.4) | 7 (0.9) |
| **10^th^ prescription, n (%)** | | 1676 (52.8) | 1359 (56.2) | 317 (41.9) |
| Time since Rx 1, weeks, median [IQR] | | 36.86 [34.14, 41.86] | 36.43 [34.00, 41.00] | 38.14 [35.00, 50.57] |
| Oral tablet | 3 mg, n (%) | 27 (0.9) | <7 | 26 (3.4) |
|  | 7 mg, n (%) | 164 (5.2) | <7 | 159 (21.0) |
|  | 14 mg, n (%) | 118 (3.7) | <7 | 116 (15.3) |
| Subcutaneous injection | 0.25 mg, n (%) | 93 (2.9) | 93 (3.8) | <7 |
|  | 0.50 mg, n (%) | 605 (19.1) | 598 (24.7) | 7 (0.9) |
|  | 1.00 mg, n (%) | 669 (21.1) | 660 (27.3) | 9 (1.2) |

| Table S10. Dose titration of semaglutide from the first prescription (Rx) to the tenth prescription, stratified by mode of administration (i.e., subcutaneous injection versus oral tablet) and body-mass index (BMI) categories*. | | | | | | | |
| --- | --- | --- | --- | --- | --- | --- | --- |
| **Semaglutide doses per prescription** | | **BMI <30 kg/m^2^ (n=508)** | | **BMI 30-<35 kg/m^2^ (n=847)** | | **BMI ≥35 kg/m^2^ (1027)** | |
|  |  | **Subcutaneous injection** | **Oral tablet** | **Subcutaneous injection** | **Oral tablet** | **Subcutaneous injection** | **Oral tablet** |
| **1^st^ prescription, n (%)** | | 327 (64.4) | 181 (35.6) | 644 (76.0) | 203 (24.0) | 849 (82.7) | 178 (17.3) |
| Time since Rx 1, weeks, median [IQR] | | 0.00 [0.00, 0.00] | 0.00 [0.00, 0.00] | 0.00 [0.00, 0.00] | 0.00 [0.00, 0.00] | 0.00 [0.00, 0.00] | 0.00 [0.00, 0.00] |
| Oral tablet | 3 mg, n (%) | <7 | 150 (82.9) | <7 | 175 (86.2) | <7 | 159 (89.3) |
|  | 7 mg, n (%) | <7 | 22 (12.2) | <7 | 22 (10.8) | <7 | 14 (7.9) |
|  | 14 mg, n (%) | <7 | 9 (5.0) | <7 | 6 (3.0) | <7 | <7 |
| Subcutaneous injection | 0.25 mg, n (%) | 252 (77.1) | <7 | 544 (84.5) | <7 | 713 (84.0) | <7 |
|  | 0.50 mg, n (%) | 62 (19.0) | <7 | 87 (13.5) | <7 | 113 (13.3) | <7 |
|  | 1.00 mg, n (%) | 13 (4.0) | <7 | 13 (2.0) | <7 | 23 (2.7) | <7 |
| **2^nd^ prescription, n (%)** | | 278 (85.0) | 148 (81.8) | 575 (89.3) | 176 (86.7) | 777 (91.5) | 156 (87.6) |
| Time since Rx 1, weeks, median [IQR] | | 4.00 [3.14, 5.43] | 4.00 [3.29, 4.71] | 3.86 [3.00, 4.93] | 4.00 [3.14, 4.86] | 4.00 [3.14, 5.00] | 4.00 [3.29, 5.00] |
| Oral tablet | 3 mg, n (%) | <7 | 37 (20.4) | <7 | 39 (19.2) | <7 | 36 (20.2) |
|  | 7 mg, n (%) | <7 | 101 (55.8) | <7 | 128 (63.1) | <7 | 112 (62.9) |
|  | 14 mg, n (%) | <7 | 10 (5.5) | <7 | 8 (3.9) | <7 | 8 (4.5) |
| Subcutaneous injection | 0.25 mg, n (%) | 77 (23.5) | <7 | 157 (24.4) | <7 | 171 (20.1) | <7 |
|  | 0.50 mg, n (%) | 174 (53.2) | <7 | 371 (57.6) | <7 | 534 (62.9) | <7 |
|  | 1.00 mg, n (%) | 26 (8.0) | <7 | 46 (7.1) | <7 | 71 (8.4) | <7 |
| **3^rd^ prescription, n (%)** | | 252 (77.1) | 128 (70.7) | 531 (82.5) | 158 (77.8) | 711 (83.7) | 136 (76.4) |
| Time since Rx 1, weeks, median [IQR] | | 8.14 [6.96, 10.18] | 8.43 [6.96, 10.04] | 8.00 [6.86, 9.57] | 8.29 [7.04, 9.14] | 8.00 [7.00, 9.57] | 8.29 [7.00, 10.75] |
| Oral tablet | 3 mg, n (%) | <7 | 30 (16.6) | <7 | 20 (9.9) | <7 | 20 (11.2) |
|  | 7 mg, n (%) | <7 | 74 (40.9) | <7 | 113 (55.7) | <7 | 91 (51.1) |
|  | 14 mg, n (%) | <7 | 24 (13.3) | <7 | 24 (11.8) | <7 | 24 (13.5) |
| Subcutaneous injection | 0.25 mg, n (%) | 42 (12.8) | <7 | 80 (12.4) | <7 | 93 (11.0) | <7 |
|  | 0.50 mg, n (%) | 148 (45.3) | <7 | 322 (50.0) | <7 | 406 (47.8) | <7 |
|  | 1.00 mg, n (%) | 61 (18.7) | <7 | 128 (19.9) | <7 | 211 (24.9) | <7 |
| **4^th^ prescription, n (%)** | | 233 (71.3) | 113 (62.4) | 490 (76.1) | 137 (67.5) | 671 (79.0) | 123 (69.1) |
| Time since Rx 1, weeks, median [IQR] | | 12.29 [10.57, 14.86] | 13.00 [11.57, 16.57] | 12.14 [10.86, 14.57] | 12.86 [11.29, 16.14] | 12.00 [10.43, 13.86] | 12.86 [10.86, 16.57] |
| Oral tablet | 3 mg, n (%) | <7 | 22 (12.2) | <7 | 13 (6.4) | <7 | 18 (10.1) |
|  | 7 mg, n (%) | <7 | 66 (36.5) | <7 | 96 (47.3) | <7 | 75 (42.1) |
|  | 14 mg, n (%) | <7 | 24 (13.3) | <7 | 26 (12.8) | <7 | 29 (16.3) |
| Subcutaneous injection | 0.25 mg, n (%) | 31 (9.5) | <7 | 60 (9.3) | <7 | 65 (7.7) | <7 |
|  | 0.50 mg, n (%) | 132 (40.4) | <7 | 268 (41.6) | <7 | 335 (39.5) | <7 |
|  | 1.00 mg, n (%) | 69 (21.1) | <7 | 160 (24.8) | <7 | 270 (31.8) | <7 |
| **5^th^ prescription, n (%)** | | 214 (65.4) | 103 (56.9) | 457 (71.0) | 129 (63.5) | 630 (74.2) | 116 (65.2) |
| Time since Rx 1, weeks, median [IQR] | | 16.57 [14.86, 19.25] | 17.71 [15.50, 22.29] | 16.29 [14.71, 19.86] | 17.57 [15.14, 22.43] | 15.86 [14.57, 18.57] | 17.00 [15.00, 22.46] |
| Oral tablet | 3 mg, n (%) | 1 (0.3) | 17 (9.4) | <7 | 12 (5.9) | <7 | 12 (6.7) |
|  | 7 mg, n (%) | <7 | 62 (34.3) | <7 | 83 (40.9) | <7 | 70 (39.3) |
|  | 14 mg, n (%) | <7 | 23 (12.7) | <7 | 30 (14.8) | <7 | 32 (18.0) |
| Subcutaneous injection | 0.25 mg, n (%) | 22 (6.7) | <7 | 44 (6.8) | <7 | 50 (5.9) | <7 |
|  | 0.50 mg, n (%) | 123 (37.6) | <7 | 245 (38.0) | <7 | 295 (34.7) | <7 |
|  | 1.00 mg, n (%) | 68 (20.8) | <7 | 167 (25.9) | <7 | 282 (33.2) | <7 |
| **6^th^ prescription, n (%)** | | 202 (61.8) | 98 (54.1) | 433 (67.2) | 118 (58.1) | 596 (70.2) | 111 (62.4) |
| Time since Rx 1, weeks, median [IQR] | | 20.79 [18.64, 23.86] | 22.07 [19.00, 28.75] | 20.29 [18.29, 24.43] | 22.43 [19.00, 28.68] | 20.00 [18.29, 22.71] | 22.00 [18.71, 28.86] |
| Oral tablet | 3 mg, n (%) | <7 | 13 (7.2) | <7 | 10 (4.9) | <7 | 15 (8.4) |
|  | 7 mg, n (%) | <7 | 60 (33.1) | <7 | 74 (36.5) | <7 | 63 (35.4) |
|  | 14 mg, n (%) | <7 | 24 (13.3) | <7 | 30 (14.8) | 1 (0.1) | 31 (17.4) |
| Subcutaneous injection | 0.25 mg, n (%) | 22 (6.7) | <7 | 34 (5.3) | <7 | 39 (4.6) | <7 |
|  | 0.50 mg, n (%) | 117 (35.8) | <7 | 226 (35.1) | <7 | 268 (31.6) | <7 |
|  | 1.00 mg, n (%) | 63 (19.3) | <7 | 171 (26.6) | <7 | 286 (33.7) | <7 |
| **7^th^ prescription, n (%)** | | 185 (56.6) | 88 (48.6) | 412 (64.0) | 111 (54.7) | 574 (67.6) | 106 (59.6) |
| Time since Rx 1, weeks, median [IQR] | | 24.71 [22.29, 28.00] | 26.21 [22.96, 32.71] | 24.43 [22.29, 28.86] | 27.71 [23.50, 35.43] | 24.00 [22.00, 27.11] | 26.36 [22.75, 34.79] |
| Oral tablet | 3 mg, n (%) | <7 | 11 (6.1) | <7 | 9 (4.4) | <7 | 11 (6.2) |
|  | 7 mg, n (%) | <7 | 54 (29.8) | <7 | 66 (32.5) | <7 | 60 (33.7) |
|  | 14 mg, n (%) | <7 | 21 (11.6) | <7 | 32 (15.8) | <7 | 33 (18.5) |
| Subcutaneous injection | 0.25 mg, n (%) | 22 (6.7) | <7 | 30 (4.7) | <7 | 38 (4.5) | <7 |
|  | 0.50 mg, n (%) | 104 (31.8) | <7 | 210 (32.6) | <7 | 248 (29.2) | <7 |
|  | 1.00 mg, n (%) | 59 (18.0) | <7 | 170 (26.4) | <7 | 286 (33.7) | <7 |
| **8^th^ prescription, n (%)** | | 177 (54.1) | 82 (45.3) | 392 (60.9) | 105 (51.7) | 551 (64.9) | 98 (55.1) |
| Time since Rx 1, weeks, median [IQR] | | 28.86 [26.43, 32.43] | 29.93 [27.04, 37.21] | 28.57 [26.14, 33.04] | 31.71 [27.57, 41.57] | 28.14 [25.86, 31.79] | 31.00 [27.14, 41.96] |
| Oral tablet | 3 mg, n (%) | <7 | 7 (3.9) | <7 | 10 (4.9) | <7 | 9 (5.1) |
|  | 7 mg, n (%) | <7 | 50 (27.6) | <7 | 54 (26.6) | <7 | 53 (29.8) |
|  | 14 mg, n (%) | <7 | 23 (12.7) | <7 | 37 (18.2) | <7 | 34 (19.1) |
| Subcutaneous injection | 0.25 mg, n (%) | 20 (6.1) | <7 | 25 (3.9) | <7 | 30 (3.5) | <7 |
|  | 0.50 mg, n (%) | 97 (29.7) | <7 | 197 (30.6) | <7 | 237 (27.9) | <7 |
|  | 1.00 mg, n (%) | 60 (18.3) | <7 | 169 (26.2) | <7 | 283 (33.3) | <7 |
| **9^th^ prescription, n (%)** | | 169 (51.7) | 77 (42.5) | 371 (57.6) | 99 (48.8) | 533 (62.8) | 90 (50.6) |
| Time since Rx 1, weeks, median [IQR] | | 32.86 [30.71, 36.29] | 34.14 [31.14, 39.00] | 32.71 [30.21, 37.71] | 35.86 [31.21, 48.00] | 32.00 [30.00, 35.86] | 34.50 [31.00, 47.14] |
| Oral tablet | 3 mg, n (%) | <7 | 8 (4.4) | <7 | 9 (4.4) | <7 | 7 (3.9) |
|  | 7 mg, n (%) | <7 | 45 (24.9) | <7 | 47 (23.2) | <7 | 47 (26.4) |
|  | 14 mg, n (%) | <7 | 22 (12.2) | <7 | 39 (19.2) | <7 | 33 (18.5) |
| Subcutaneous injection | 0.25 mg, n (%) | 17 (5.2) | <7 | 22 (3.4) | <7 | 32 (3.8) | <7 |
|  | 0.50 mg, n (%) | 94 (28.7) | <7 | 179 (27.8) | <7 | 218 (25.7) | <7 |
|  | 1.00 mg, n (%) | 58 (17.7) | <7 | 170 (26.4) | <7 | 281 (33.1) | <7 |
| **10^th^ prescription, n (%)** | | 158 (48.3) | 68 (37.6) | 352 (54.7) | 84 (41.4) | 511 (60.2) | 85 (47.8) |
| Time since Rx 1, weeks, median [IQR] | | 36.71 [34.14, 40.00] | 37.79 [35.00, 41.89] | 36.71 [34.14, 42.04] | 38.21 [35.07, 48.86] | 36.14 [34.00, 40.14] | 37.57 [34.86, 51.29] |
| Oral tablet | 3 mg, n (%) | <7 | <7 | <7 | 7 (3.4) | <7 | <7 |
|  | 7 mg, n (%) | <7 | 42 (23.2) | <7 | 39 (19.2) | <7 | 43 (24.2) |
|  | 14 mg, n (%) | <7 | 19 (10.5) | <7 | 35 (17.2) | <7 | 33 (18.5) |
| Subcutaneous injection | 0.25 mg, n (%) | 15 (4.6) | <7 | 23 (3.6) | <7 | 28 (3.3) | <7 |
|  | 0.50 mg, n (%) | 92 (28.1) | <7 | 168 (26.1) | <7 | 205 (24.1) | <7 |
|  | 1.00 mg, n (%) | 51 (15.6) | <7 | 160 (24.8) | <7 | 275 (32.4) | <7 |
| *Based on the most recent measurement recorded within the year before or at semaglutide initiation. | | | | | | | |

| Table S11. Dose titration of semaglutide from the first prescription (Rx) to the tenth prescription, stratified by mode of administration (i.e., subcutaneous injection versus oral tablet) and cardiovascular disease (CVD) history. | | | | | |
| --- | --- | --- | --- | --- | --- |
| **Semaglutide doses per prescription** | | **CVD history** | | **No CVD history** | |
|  |  | **Subcutaneous injection** | **Oral tablet** | **Subcutaneous injection** | **Oral tablet** |
| **1^st^ prescription, n (%)** | | 424 (100.0) | 145 (100.0) | 1993 (100.0) | 611 (100.0) |
| Time since Rx 1, weeks, median [IQR] | | 0.00 [0.00, 0.00] | 0.00 [0.00, 0.00] | 0.00 [0.00, 0.00] | 0.00 [0.00, 0.00] |
| Oral tablet | 3 mg, n (%) | <7 | 131 (90.3) | <7 | 524 (85.8) |
|  | 7 mg, n (%) | <7 | 13 (9.0) | <7 | 63 (10.3) |
|  | 14 mg, n (%) | <7 | <7 | <7 | 24 (3.9) |
| Subcutaneous injection | 0.25 mg, n (%) | 361 (85.1) | <7 | 1636 (82.1) | <7 |
|  | 0.50 mg, n (%) | 54 (12.7) | <7 | 291 (14.6) | <7 |
|  | 1.00 mg, n (%) | 9 (2.1) | <7 | 66 (3.3) | <7 |
| **2^nd^ prescription, n (%)** | | 362 (85.4) | 118 (81.4) | 1791 (89.9) | 521 (85.3) |
| Time since Rx 1, weeks, median [IQR] | | 4.00 [3.00, 5.00] | 4.00 [3.18, 4.82] | 4.00 [3.14, 5.14] | 4.00 [3.14, 5.00] |
| Oral tablet | 3 mg, n (%) | <7 | 33 (22.8) | <7 | 129 (21.1) |
|  | 7 mg, n (%) | <7 | 81 (55.9) | <7 | 359 (58.8) |
|  | 14 mg, n (%) | <7 | <7 | <7 | 31 (5.1) |
| Subcutaneous injection | 0.25 mg, n (%) | 97 (22.9) | <7 | 433 (21.7) | <7 |
|  | 0.50 mg, n (%) | 243 (57.3) | <7 | 1177 (59.1) | <7 |
|  | 1.00 mg, n (%) | 22 (5.2) | <7 | 176 (8.8) | <7 |
| **3^rd^ prescription, n (%)** | | 325 (76.7) | 106 (73.1) | 1663 (83.4) | 466 (76.3) |
| Time since Rx 1, weeks, median [IQR] | | 8.00 [6.57, 10.00] | 8.57 [7.00, 9.96] | 8.00 [7.00, 9.71] | 8.29 [7.00, 10.71] |
| Oral tablet | 3 mg, n (%) | <7 | 23 (15.9) | <7 | 85 (13.9) |
|  | 7 mg, n (%) | <7 | 67 (46.2) | <7 | 301 (49.3) |
|  | 14 mg, n (%) | <7 | 14 (9.7) | <7 | 76 (12.4) |
| Subcutaneous injection | 0.25 mg, n (%) | 59 (13.9) | <7 | 237 (11.9) | <7 |
|  | 0.50 mg, n (%) | 187 (44.1) | <7 | 963 (48.3) | <7 |
|  | 1.00 mg, n (%) | 78 (18.4) | <7 | 460 (23.1) | <7 |
| **4^th^ prescription, n (%)** | | 297 (70.0) | 96 (66.2) | 1550 (77.8) | 410 (67.1) |
| Time since Rx 1, weeks, median [IQR] | | 12.00 [10.14, 14.00] | 12.50 [10.64, 16.29] | 12.00 [10.57, 14.29] | 12.86 [11.18, 16.57] |
| Oral tablet | 3 mg, n (%) | <7 | 15 (10.3) | <7 | 61 (10.0) |
|  | 7 mg, n (%) | <7 | 64 (44.1) | <7 | 258 (42.2) |
|  | 14 mg, n (%) | <7 | 14 (9.7) | <7 | 86 (14.1) |
| Subcutaneous injection | 0.25 mg, n (%) | 36 (8.5) | <7 | 182 (9.1) | <7 |
|  | 0.50 mg, n (%) | 158 (37.3) | <7 | 792 (39.7) | <7 |
|  | 1.00 mg, n (%) | 102 (24.1) | <7 | 571 (28.7) | <7 |
| **5^th^ prescription, n (%)** | | 271 (63.9) | 89 (61.4) | 1453 (72.9) | 381 (62.4) |
| Time since Rx 1, weeks, median [IQR] | | 16.14 [14.29, 18.64] | 17.43 [14.86, 22.14] | 16.14 [14.57, 19.14] | 17.14 [15.14, 23.00] |
| Oral tablet | 3 mg, n (%) | <7 | 11 (7.6) | <7 | 49 (8.0) |
|  | 7 mg, n (%) | <7 | 59 (40.7) | <7 | 230 (37.6) |
|  | 14 mg, n (%) | <7 | 16 (11.0) | <7 | 95 (15.5) |
| Subcutaneous injection | 0.25 mg, n (%) | 27 (6.4) | <7 | 134 (6.7) | <7 |
|  | 0.50 mg, n (%) | 133 (31.4) | <7 | 724 (36.3) | <7 |
|  | 1.00 mg, n (%) | 110 (25.9) | <7 | 589 (29.6) | <7 |
| **6^th^ prescription, n (%)** | | 255 (60.1) | 81 (55.9) | 1380 (69.2) | 357 (58.4) |
| Time since Rx 1, weeks, median [IQR] | | 20.14 [18.07, 22.93] | 21.86 [18.43, 28.00] | 20.14 [18.29, 23.57] | 22.71 [19.00, 29.43] |
| Oral tablet | 3 mg, n (%) | <7 | 8 (5.5) | <7 | 45 (7.4) |
|  | 7 mg, n (%) | <7 | 52 (35.9) | <7 | 207 (33.9) |
|  | 14 mg, n (%) | <7 | 18 (12.4) | <7 | 95 (15.5) |
| Subcutaneous injection | 0.25 mg, n (%) | 27 (6.4) | <7 | 107 (5.4) | <7 |
|  | 0.50 mg, n (%) | 111 (26.2) | <7 | 680 (34.1) | <7 |
|  | 1.00 mg, n (%) | 115 (27.1) | <7 | 587 (29.5) | <7 |
| **7^th^ prescription, n (%)** | | 250 (59.0) | 71 (49.0) | 1305 (65.5) | 336 (55.0) |
| Time since Rx 1, weeks, median [IQR] | | 24.21 [22.00, 27.11] | 26.14 [22.71, 33.86] | 24.29 [22.14, 28.00] | 26.86 [23.29, 35.18] |
| Oral tablet | 3 mg, n (%) | <7 | 7 (4.8) | <7 | 34 (5.6) |
|  | 7 mg, n (%) | <7 | 44 (30.3) | <7 | 190 (31.1) |
|  | 14 mg, n (%) | <7 | 18 (12.4) | <7 | 100 (16.4) |
| Subcutaneous injection | 0.25 mg, n (%) | 25 (5.9) | <7 | 103 (5.2) | <7 |
|  | 0.50 mg, n (%) | 110 (25.9) | <7 | 615 (30.9) | <7 |
|  | 1.00 mg, n (%) | 114 (26.9) | <7 | 582 (29.2) | <7 |
| **8^th^ prescription, n (%)** | | 238 (56.1) | 66 (45.5) | 1245 (62.5) | 310 (50.7) |
| Time since Rx 1, weeks, median [IQR] | | 28.36 [26.00, 32.11] | 30.64 [26.71, 39.61] | 28.29 [26.14, 32.29] | 30.86 [27.29, 41.00] |
| Oral tablet | 3 mg, n (%) | <7 | 7 (4.8) | <7 | 30 (4.9) |
|  | 7 mg, n (%) | <7 | 35 (24.1) | <7 | 165 (27.0) |
|  | 14 mg, n (%) | <7 | 22 (15.2) | <7 | 102 (16.7) |
| Subcutaneous injection | 0.25 mg, n (%) | 21 (5.0) | <7 | 87 (4.4) | <7 |
|  | 0.50 mg, n (%) | 103 (24.3) | <7 | 574 (28.8) | <7 |
|  | 1.00 mg, n (%) | 114 (26.9) | <7 | 580 (29.1) | <7 |
| **9^th^ prescription, n (%)** | | 227 (53.5) | 62 (42.8) | 1194 (59.9) | 288 (47.1) |
| Time since Rx 1, weeks, median [IQR] | | 32.43 [30.00, 36.07] | 34.07 [30.57, 47.00] | 32.43 [30.14, 36.71] | 35.21 [31.43, 46.86] |
| Oral tablet | 3 mg, n (%) | <7 | 6 (4.1) | <7 | 27 (4.4) |
|  | 7 mg, n (%) | <7 | 33 (22.8) | <7 | 144 (23.6) |
|  | 14 mg, n (%) | <7 | 21 (14.5) | <7 | 102 (16.7) |
| Subcutaneous injection | 0.25 mg, n (%) | 15 (3.5) | <7 | 83 (4.2) | <7 |
|  | 0.50 mg, n (%) | 97 (22.9) | <7 | 535 (26.8) | <7 |
|  | 1.00 mg, n (%) | 115 (27.1) | <7 | 572 (28.7) | <7 |
| **10^th^ prescription, n (%)** | | 223 (52.6) | 57 (39.3) | 1136 (57.0) | 260 (42.6) |
| Time since Rx 1, weeks, median [IQR] | | 36.71 [33.86, 41.00] | 38.43 [35.00, 51.00] | 36.43 [34.14, 40.89] | 37.93 [35.14, 49.50] |
| Oral tablet | 3 mg, n (%) | <7 | 4 (2.8) | <7 | 22 (3.6) |
|  | 7 mg, n (%) | <7 | 29 (20.0) | <7 | 130 (21.3) |
|  | 14 mg, n (%) | <7 | 22 (15.2) | <7 | 94 (15.4) |
| Subcutaneous injection | 0.25 mg, n (%) | 15 (3.5) | <7 | 78 (3.9) | <7 |
|  | 0.50 mg, n (%) | 98 (23.1) | <7 | 500 (25.1) | <7 |
|  | 1.00 mg, n (%) | 108 (25.5) | <7 | 552 (27.7) | 8 (1.3) |

| Table S12. Dose titration of semaglutide from the first prescription (Rx) to the tenth prescription, stratified by mode of administration (i.e., subcutaneous injection versus oral tablet) and sex assigned at birth. | | | | | |
| --- | --- | --- | --- | --- | --- |
| **Semaglutide doses per prescription** | | **Female** | | **Male** | |
|  |  | **Subcutaneous injection** | **Oral tablet** | **Subcutaneous injection** | **Oral tablet** |
| **1^st^ prescription, n (%)** | | 1091 (100.0) | 290 (100.0) | 1326 (100.0) | 466 (100.0) |
| Time since Rx 1, weeks, median [IQR] | | 0.00 [0.00, 0.00] | 0.00 [0.00, 0.00] | 0.00 [0.00, 0.00] | 0.00 [0.00, 0.00] |
| Oral tablet | 3 mg, n (%) | <7 | 252 (86.9) | <7 | 403 (86.5) |
|  | 7 mg, n (%) | <7 | 28 (9.7) | <7 | 48 (10.3) |
|  | 14 mg, n (%) | <7 | 10 (3.4) | <7 | 15 (3.2) |
| Subcutaneous injection | 0.25 mg, n (%) | 913 (83.7) | <7 | 1084 (81.7) | <7 |
|  | 0.50 mg, n (%) | 151 (13.8) | <7 | 194 (14.6) | <7 |
|  | 1.00 mg, n (%) | 27 (2.5) | <7 | 48 (3.6) | <7 |
| **2^nd^ prescription, n (%)** | | 972 (89.1) | 246 (84.8) | 1181 (89.1) | 393 (84.3) |
| Time since Rx 1, weeks, median [IQR] | | 4.00 [3.00, 5.00] | 3.86 [3.14, 4.71] | 4.00 [3.14, 5.14] | 4.00 [3.29, 5.00] |
| Oral tablet | 3 mg, n (%) | <7 | 62 (21.4) | <7 | 100 (21.5) |
|  | 7 mg, n (%) | <7 | 172 (59.3) | <7 | 268 (57.5) |
|  | 14 mg, n (%) | <7 | 12 (4.1) | <7 | 22 (4.7) |
| Subcutaneous injection | 0.25 mg, n (%) | 248 (22.7) | <7 | 282 (21.3) | <7 |
|  | 0.50 mg, n (%) | 646 (59.2) | <7 | 774 (58.4) | <7 |
|  | 1.00 mg, n (%) | 75 (6.9) | <7 | 123 (9.3) | <7 |
| **3^rd^ prescription, n (%)** | | 895 (82.0) | 221 (76.2) | 1093 (82.4) | 351 (75.3) |
| Time since Rx 1, weeks, median [IQR] | | 8.00 [6.86, 9.50] | 8.29 [7.00, 10.57] | 8.00 [7.00, 10.00] | 8.29 [7.00, 10.50] |
| Oral tablet | 3 mg, n (%) | <7 | 42 (14.5) | <7 | 66 (14.2) |
|  | 7 mg, n (%) | <7 | 148 (51.0) | <7 | 220 (47.2) |
|  | 14 mg, n (%) | <7 | 30 (10.3) | <7 | 60 (12.9) |
| Subcutaneous injection | 0.25 mg, n (%) | 138 (12.6) | <7 | 158 (11.9) | <7 |
|  | 0.50 mg, n (%) | 545 (50.0) | <7 | 605 (45.6) | <7 |
|  | 1.00 mg, n (%) | 210 (19.2) | <7 | 328 (24.7) | <7 |
| **4^th^ prescription, n (%)** | | 832 (76.3) | 195 (67.2) | 1015 (76.5) | 311 (66.7) |
| Time since Rx 1, weeks, median [IQR] | | 12.00 [10.43, 14.00] | 12.71 [11.00, 16.29] | 12.14 [10.71, 14.71] | 12.86 [11.14, 16.71] |
| Oral tablet | 3 mg, n (%) | <7 | 26 (9.0) | <7 | 50 (10.7) |
|  | 7 mg, n (%) | <7 | 128 (44.1) | <7 | 194 (41.6) |
|  | 14 mg, n (%) | <7 | 39 (13.4) | <7 | 61 (13.1) |
| Subcutaneous injection | 0.25 mg, n (%) | 109 (10.0) | <7 | 109 (8.2) | <7 |
|  | 0.50 mg, n (%) | 420 (38.5) | <7 | 530 (40.0) | <7 |
|  | 1.00 mg, n (%) | 300 (27.5) | <7 | 373 (28.1) | <7 |
| **5^th^ prescription, n (%)** | | 772 (70.8) | 183 (63.1) | 952 (71.8) | 287 (61.6) |
| Time since Rx 1, weeks, median [IQR] | | 16.00 [14.43, 18.57] | 17.43 [15.00, 23.07] | 16.29 [14.57, 19.43] | 17.14 [15.14, 22.29] |
| Oral tablet | 3 mg, n (%) | <7 | 19 (6.6) | <7 | 41 (8.8) |
|  | 7 mg, n (%) | <7 | 118 (40.7) | <7 | 171 (36.7) |
|  | 14 mg, n (%) | <7 | 42 (14.5) | <7 | 69 (14.8) |
| Subcutaneous injection | 0.25 mg, n (%) | 76 (7.0) | <7 | 85 (6.4) | <7 |
|  | 0.50 mg, n (%) | 377 (34.6) | <7 | 480 (36.2) | <7 |
|  | 1.00 mg, n (%) | 316 (29.0) | <7 | 383 (28.9) | <7 |
| **6^th^ prescription, n (%)** | | 732 (67.1) | 169 (58.3) | 903 (68.1) | 269 (57.7) |
| Time since Rx 1, weeks, median [IQR] | | 20.00 [18.14, 23.04] | 22.00 [19.14, 28.14] | 20.14 [18.29, 23.86] | 22.14 [18.86, 29.14] |
| Oral tablet | 3 mg, n (%) | <7 | 17 (5.9) | <7 | 36 (7.7) |
|  | 7 mg, n (%) | <7 | 105 (36.2) | <7 | 154 (33.0) |
|  | 14 mg, n (%) | <7 | 43 (14.8) | <7 | 70 (15.0) |
| Subcutaneous injection | 0.25 mg, n (%) | 63 (5.8) | <7 | 71 (5.4) | <7 |
|  | 0.50 mg, n (%) | 343 (31.4) | <7 | 448 (33.8) | <7 |
|  | 1.00 mg, n (%) | 322 (29.5) | <7 | 380 (28.7) | <7 |
| **7^th^ prescription, n (%)** | | 696 (63.8) | 159 (54.8) | 859 (64.8) | 248 (53.2) |
| Time since Rx 1, weeks, median [IQR] | | 24.07 [21.86, 27.14] | 26.14 [23.21, 34.79] | 24.43 [22.43, 28.29] | 26.86 [23.11, 35.18] |
| Oral tablet | 3 mg, n (%) | <7 | 17 (5.9) | <7 | 24 (5.2) |
|  | 7 mg, n (%) | <7 | 96 (33.1) | <7 | 138 (29.6) |
|  | 14 mg, n (%) | <7 | 43 (14.8) | <7 | 75 (16.1) |
| Subcutaneous injection | 0.25 mg, n (%) | 63 (5.8) | <7 | 65 (4.9) | <7 |
|  | 0.50 mg, n (%) | 319 (29.2) | <7 | 406 (30.6) | <7 |
|  | 1.00 mg, n (%) | 310 (28.4) | <7 | 386 (29.1) | <7 |
| **8^th^ prescription, n (%)** | | 663 (60.8) | 142 (49.0) | 820 (61.8) | 234 (50.2) |
| Time since Rx 1, weeks, median [IQR] | | 28.14 [25.86, 31.79] | 30.64 [26.93, 41.07] | 28.71 [26.29, 33.14] | 30.93 [27.32, 41.00] |
| Oral tablet | 3 mg, n (%) | <7 | 17 (5.9) | <7 | 20 (4.3) |
|  | 7 mg, n (%) | <7 | 78 (26.9) | <7 | 122 (26.2) |
|  | 14 mg, n (%) | <7 | 43 (14.8) | <7 | 81 (17.4) |
| Subcutaneous injection | 0.25 mg, n (%) | 54 (4.9) | <7 | 54 (4.1) | <7 |
|  | 0.50 mg, n (%) | 291 (26.7) | <7 | 386 (29.1) | <7 |
|  | 1.00 mg, n (%) | 315 (28.9) | <7 | 379 (28.6) | <7 |
| **9^th^ prescription, n (%)** | | 633 (58.0) | 134 (46.2) | 788 (59.4) | 216 (46.4) |
| Time since Rx 1, weeks, median [IQR] | | 32.29 [30.14, 35.86] | 34.50 [31.07, 47.07] | 32.57 [30.14, 37.14] | 35.36 [31.14, 46.86] |
| Oral tablet | 3 mg, n (%) | <7 | 16 (5.5) | <7 | 17 (3.6) |
|  | 7 mg, n (%) | <7 | 71 (24.5) | <7 | 106 (22.7) |
|  | 14 mg, n (%) | <7 | 43 (14.8) | <7 | 80 (17.2) |
| Subcutaneous injection | 0.25 mg, n (%) | 49 (4.5) | <7 | 49 (3.7) | <7 |
|  | 0.50 mg, n (%) | 267 (24.5) | <7 | 365 (27.5) | <7 |
|  | 1.00 mg, n (%) | 315 (28.9) | <7 | 372 (28.1) | <7 |
| **10^th^ prescription, n (%)** | | 607 (55.6) | 119 (41.0) | 752 (56.7) | 198 (42.5) |
| Time since Rx 1, weeks, median [IQR] | | 36.14 [33.86, 40.00] | 37.43 [34.86, 48.36] | 36.79 [34.14, 41.71] | 38.64 [35.14, 52.46] |
| Oral tablet | 3 mg, n (%) | <7 | 12 (4.1) | <7 | 14 (3.0) |
|  | 7 mg, n (%) | <7 | 65 (22.4) | <7 | 94 (20.2) |
|  | 14 mg, n (%) | <7 | 39 (13.4) | <7 | 77 (16.5) |
| Subcutaneous injection | 0.25 mg, n (%) | 47 (4.3) | <7 | 46 (3.5) | <7 |
|  | 0.50 mg, n (%) | 257 (23.6) | <7 | 341 (25.7) | <7 |
|  | 1.00 mg, n (%) | 298 (27.3) | <7 | 362 (27.3) | 8 (1.7) |

| Table S13. Dose titration of dulaglutide from the first prescription (Rx) to the tenth prescription, overall and stratified by body-mass index (BMI) categories* | | | | | |
| --- | --- | --- | --- | --- | --- |
| **Dulaglutide doses per prescription** | | **Dulaglutide overall** | **BMI <30 kg/m^2^** | **BMI 30-<35 kg/m^2^** | **BMI ≥35 kg/m^2^** |
| **1^st^ prescription, n (%)** | | 3812 (100.0) | 619 (16.3) | 1076 (28.2) | 1231 (32.3) |
| Time since Rx 1, weeks, median [IQR] | | 0.00 [0.00, 0.00] | 0.00 [0.00, 0.00] | 0.00 [0.00, 0.00] | 0.00 [0.00, 0.00] |
| Subcutaneous injection | 0.75 mg, n (%) | 1765 (46.3) | 301 (48.6) | 485 (45.1) | 566 (46.0) |
|  | 1.50 mg, n (%) | 2041 (53.5) | 317 (51.2) | 589 (54.7) | 665 (54.0) |
|  | 3.00 mg, n (%) | <7 | <7 | <7 | <7 |
|  | 4.50 mg, n (%) | <7 | <7 | <7 | <7 |
| **2^nd^ prescription, n (%)** | | 3488 (91.5) | 545 (88.0) | 988 (91.8) | 1150 (93.4) |
| Time since Rx 1, weeks, median [IQR] | | 3.71 [2.86, 4.43] | 3.71 [3.00, 4.57] | 3.71 [2.86, 4.43] | 3.57 [2.86, 4.29] |
| Subcutaneous injection | 0.75 mg, n (%) | 1167 (30.6) | 206 (33.3) | 323 (30.0) | 371 (30.1) |
|  | 1.50 mg, n (%) | 2296 (60.2) | 336 (54.3) | 659 (61.2) | 773 (62.8) |
|  | 3.00 mg, n (%) | 22 (0.6) | <7 | <7 | <7 |
|  | 4.50 mg, n (%) | <7 | <7 | <7 | <7 |
| **3^rd^ prescription, n (%)** | | 3309 (86.8) | 511 (82.6) | 937 (87.1) | 1102 (89.5) |
| Time since Rx 1, weeks, median [IQR] | | 7.71 [6.57, 8.86] | 7.71 [6.57, 9.29] | 7.71 [6.71, 8.86] | 7.57 [6.43, 8.57] |
| Subcutaneous injection | 0.75 mg, n (%) | 970 (25.4) | 174 (28.1) | 282 (26.2) | 312 (25.3) |
|  | 1.50 mg, n (%) | 2288 (60.0) | 332 (53.6) | 638 (59.3) | 775 (63.0) |
|  | 3.00 mg, n (%) | 44 (1.2) | <7 | 14 (1.3) | 13 (1.1) |
|  | 4.50 mg, n (%) | <7 | <7 | <7 | <7 |
| **4^th^ prescription, n (%)** | | 3159 (82.9) | 479 (77.4) | 895 (83.2) | 1059 (86.0) |
| Time since Rx 1, weeks, median [IQR] | | 11.71 [10.43, 13.29] | 11.86 [10.29, 13.86] | 11.71 [10.57, 13.43] | 11.57 [10.29, 12.86] |
| Subcutaneous injection | 0.75 mg, n (%) | 859 (22.5) | 148 (23.9) | 254 (23.6) | 281 (22.8) |
|  | 1.50 mg, n (%) | 2218 (58.2) | 322 (52.0) | 619 (57.5) | 751 (61.0) |
|  | 3.00 mg, n (%) | 69 (1.8) | 9 (1.5) | 17 (1.6) | 23 (1.9) |
|  | 4.50 mg, n (%) | 13 (0.3) | <7 | <7 | <7 |
| **5^th^ prescription, n (%)** | | 3027 (79.4) | 447 (72.2) | 865 (80.4) | 1022 (83.0) |
| Time since Rx 1, weeks, median [IQR] | | 15.71 [14.14, 17.71] | 15.71 [14.29, 18.00] | 15.86 [14.43, 17.86] | 15.71 [14.14, 17.29] |
| Subcutaneous injection | 0.75 mg, n (%) | 755 (19.8) | 123 (19.9) | 225 (20.9) | 260 (21.1) |
|  | 1.50 mg, n (%) | 2159 (56.6) | 315 (50.9) | 614 (57.1) | 722 (58.7) |
|  | 3.00 mg, n (%) | 97 (2.5) | 8 (1.3) | 21 (2.0) | 35 (2.8) |
|  | 4.50 mg, n (%) | 16 (0.4) | <7 | <7 | <7 |
| **6^th^ prescription, n (%)** | | 2916 (76.5) | 426 (68.8) | 834 (77.5) | 996 (80.9) |
| Time since Rx 1, weeks, median [IQR] | | 19.71 [18.14, 22.00] | 19.71 [18.14, 22.54] | 19.79 [18.29, 22.14] | 19.71 [18.00, 21.61] |
| Subcutaneous injection | 0.75 mg, n (%) | 690 (18.1) | 120 (19.4) | 199 (18.5) | 233 (18.9) |
|  | 1.50 mg, n (%) | 2091 (54.9) | 291 (47.0) | 603 (56.0) | 714 (58.0) |
|  | 3.00 mg, n (%) | 115 (3.0) | 13 (2.1) | 24 (2.2) | 44 (3.6) |
|  | 4.50 mg, n (%) | 20 (0.5) | <7 | 8 (0.7) | <7 |
| **7^th^ prescription, n (%)** | | 2796 (73.3) | 405 (65.4) | 801 (74.4) | 956 (77.7) |
| Time since Rx 1, weeks, median [IQR] | | 23.86 [22.00, 26.43] | 23.71 [22.00, 26.86] | 23.86 [22.29, 26.71] | 23.57 [22.00, 26.00] |
| Subcutaneous injection | 0.75 mg, n (%) | 626 (16.4) | 105 (17.0) | 176 (16.4) | 214 (17.4) |
|  | 1.50 mg, n (%) | 2018 (52.9) | 285 (46.0) | 587 (54.6) | 686 (55.7) |
|  | 3.00 mg, n (%) | 123 (3.2) | 13 (2.1) | 28 (2.6) | 46 (3.7) |
|  | 4.50 mg, n (%) | 29 (0.8) | <7 | 10 (0.9) | 10 (0.8) |
| **8^th^ prescription, n (%)** | | 2689 (70.5) | 385 (62.2) | 769 (71.5) | 933 (75.8) |
| Time since Rx 1, weeks, median [IQR] | | 27.86 [26.00, 30.86] | 27.86 [26.14, 31.14] | 27.71 [26.00, 31.29] | 27.71 [26.00, 30.00] |
| Subcutaneous injection | 0.75 mg, n (%) | 581 (15.2) | 100 (16.2) | 161 (15.0) | 200 (16.2) |
|  | 1.50 mg, n (%) | 1947 (51.1) | 269 (43.5) | 569 (52.9) | 667 (54.2) |
|  | 3.00 mg, n (%) | 129 (3.4) | 14 (2.3) | 28 (2.6) | 54 (4.4) |
|  | 4.50 mg, n (%) | 32 (0.8) | <7 | 11 (1.0) | 12 (1.0) |
| **9^th^ prescription, n (%)** | | 2587 (67.9) | 370 (59.8) | 742 (69.0) | 890 (72.3) |
| Time since Rx 1, weeks, median [IQR] | | 31.86 [30.00, 34.86] | 31.86 [29.89, 35.25] | 32.00 [30.14, 35.39] | 31.71 [29.86, 34.14] |
| Subcutaneous injection | 0.75 mg, n (%) | 547 (14.3) | 96 (15.5) | 151 (14.0) | 185 (15.0) |
|  | 1.50 mg, n (%) | 1861 (48.8) | 255 (41.2) | 548 (50.9) | 639 (51.9) |
|  | 3.00 mg, n (%) | 149 (3.9) | 16 (2.6) | 34 (3.2) | 54 (4.4) |
|  | 4.50 mg, n (%) | 30 (0.8) | <7 | 9 (0.8) | 12 (1.0) |
| **10^th^ prescription, n (%)** | | 2474 (64.9) | 352 (56.9) | 713 (66.3) | 855 (69.5) |
| Time since Rx 1, weeks, median [IQR] | | 35.86 [33.89, 39.00] | 35.79 [33.57, 39.04] | 35.86 [34.00, 40.14] | 35.71 [33.86, 38.29] |
| Subcutaneous injection | 0.75 mg, n (%) | 505 (13.2) | 92 (14.9) | 138 (12.8) | 174 (14.1) |
|  | 1.50 mg, n (%) | 1769 (46.4) | 238 (38.4) | 518 (48.1) | 614 (49.9) |
|  | 3.00 mg, n (%) | 165 (4.3) | 18 (2.9) | 47 (4.4) | 54 (4.4) |
|  | 4.50 mg, n (%) | 35 (0.9) | <7 | 10 (0.9) | 13 (1.1) |
| *Based on the most recent measurement recorded within the year before or at dulaglutide initiation. | | | | | |

| Table S14. Dose titration of dulaglutide from the first prescription (Rx) to the tenth prescription, overall and by subgroups of cardiovascular disease (CVD) history and sex assigned at birth. | | | | | | |
| --- | --- | --- | --- | --- | --- | --- |
| **Dulaglutide doses per prescription** | | **Dulaglutide overall** | **CVD history** | **No CVD history** | **Female** | **Male** |
| **1^st^ prescription, n (%)** | | 3812 (100.0) | 738 (100.0) | 3074 (100.0) | 1729 (100.0) | 2083 (100.0) |
| Time since Rx 1, weeks, median [IQR] | | 0.00 [0.00, 0.00] | 0.00 [0.00, 0.00] | 0.00 [0.00, 0.00] | 0.00 [0.00, 0.00] | 0.00 [0.00, 0.00] |
| Subcutaneous injection | 0.75 mg, n (%) | 1765 (46.3) | 365 (49.5) | 1400 (45.5) | 835 (48.3) | 930 (44.6) |
|  | 1.50 mg, n (%) | 2041 (53.5) | 372 (50.4) | 1669 (54.3) | 890 (51.5) | 1151 (55.3) |
|  | 3.00 mg, n (%) | <7 | <7 | <7 | <7 | <7 |
|  | 4.50 mg, n (%) | <7 | <7 | <7 | <7 | <7 |
| **2^nd^ prescription, n (%)** | | 3488 (91.5) | 669 (90.7) | 2819 (91.7) | 1574 (91.0) | 1914 (91.9) |
| Time since Rx 1, weeks, median [IQR] | | 3.71 [2.86, 4.43] | 3.71 [3.00, 4.57] | 3.71 [2.86, 4.43] | 3.57 [2.86, 4.29] | 3.71 [2.86, 4.57] |
| Subcutaneous injection | 0.75 mg, n (%) | 1167 (30.6) | 251 (34.0) | 916 (29.8) | 548 (31.7) | 619 (29.7) |
|  | 1.50 mg, n (%) | 2296 (60.2) | 412 (55.8) | 1884 (61.3) | 1016 (58.8) | 1280 (61.4) |
|  | 3.00 mg, n (%) | 22 (0.6) | <7 | 17 (0.6) | 9 (0.5) | 13 (0.6) |
|  | 4.50 mg, n (%) | <7 | <7 | <7 | <7 | <7 |
| **3^rd^ prescription, n (%)** | | 3309 (86.8) | 638 (86.4) | 2671 (86.9) | 1500 (86.8) | 1809 (86.8) |
| Time since Rx 1, weeks, median [IQR] | | 7.71 [6.57, 8.86] | 7.86 [6.61, 9.00] | 7.71 [6.57, 8.86] | 7.57 [6.29, 8.86] | 7.71 [6.71, 9.00] |
| Subcutaneous injection | 0.75 mg, n (%) | 970 (25.4) | 215 (29.1) | 755 (24.6) | 455 (26.3) | 515 (24.7) |
|  | 1.50 mg, n (%) | 2288 (60.0) | 410 (55.6) | 1878 (61.1) | 1022 (59.1) | 1266 (60.8) |
|  | 3.00 mg, n (%) | 44 (1.2) | 11 (1.5) | 33 (1.1) | 19 (1.1) | 25 (1.2) |
|  | 4.50 mg, n (%) | <7 | <7 | <7 | <7 | <7 |
| **4^th^ prescription, n (%)** | | 3159 (82.9) | 610 (82.7) | 2549 (82.9) | 1432 (82.8) | 1727 (82.9) |
| Time since Rx 1, weeks, median [IQR] | | 11.71 [10.43, 13.29] | 11.86 [10.57, 13.71] | 11.71 [10.29, 13.14] | 11.57 [10.14, 13.00] | 11.86 [10.50, 13.57] |
| Subcutaneous injection | 0.75 mg, n (%) | 859 (22.5) | 194 (26.3) | 665 (21.6) | 411 (23.8) | 448 (21.5) |
|  | 1.50 mg, n (%) | 2218 (58.2) | 392 (53.1) | 1826 (59.4) | 979 (56.6) | 1239 (59.5) |
|  | 3.00 mg, n (%) | 69 (1.8) | 20 (2.7) | 49 (1.6) | 34 (2.0) | 35 (1.7) |
|  | 4.50 mg, n (%) | 13 (0.3) | <7 | 9 (0.3) | 8 (0.5) | <7 |
| **5^th^ prescription, n (%)** | | 3027 (79.4) | 582 (78.9) | 2445 (79.5) | 1376 (79.6) | 1651 (79.3) |
| Time since Rx 1, weeks, median [IQR] | | 15.71 [14.14, 17.71] | 15.86 [14.43, 18.00] | 15.71 [14.14, 17.71] | 15.57 [14.14, 17.71] | 15.86 [14.43, 17.86] |
| Subcutaneous injection | 0.75 mg, n (%) | 755 (19.8) | 167 (22.6) | 588 (19.1) | 369 (21.3) | 386 (18.5) |
|  | 1.50 mg, n (%) | 2159 (56.6) | 388 (52.6) | 1771 (57.6) | 952 (55.1) | 1207 (57.9) |
|  | 3.00 mg, n (%) | 97 (2.5) | 22 (3.0) | 75 (2.4) | 46 (2.7) | 51 (2.4) |
|  | 4.50 mg, n (%) | 16 (0.4) | <7 | 11 (0.4) | 9 (0.5) | 7 (0.3) |
| **6^th^ prescription, n (%)** | | 2916 (76.5) | 560 (75.9) | 2356 (76.6) | 1327 (76.7) | 1589 (76.3) |
| Time since Rx 1, weeks, median [IQR] | | 19.71 [18.14, 22.00] | 19.86 [18.29, 22.14] | 19.71 [18.00, 22.00] | 19.71 [17.86, 22.00] | 19.86 [18.43, 22.29] |
| Subcutaneous injection | 0.75 mg, n (%) | 690 (18.1) | 151 (20.5) | 539 (17.5) | 339 (19.6) | 351 (16.9) |
|  | 1.50 mg, n (%) | 2091 (54.9) | 378 (51.2) | 1713 (55.7) | 920 (53.2) | 1171 (56.2) |
|  | 3.00 mg, n (%) | 115 (3.0) | 24 (3.3) | 91 (3.0) | 58 (3.4) | 57 (2.7) |
|  | 4.50 mg, n (%) | 20 (0.5) | 7 (0.9) | 13 (0.4) | 10 (0.6) | 10 (0.5) |
| **7^th^ prescription, n (%)** | | 2796 (73.3) | 536 (72.6) | 2260 (73.5) | 1276 (73.8) | 1520 (73.0) |
| Time since Rx 1, weeks, median [IQR] | | 23.86 [22.00, 26.43] | 23.86 [22.14, 26.86] | 23.86 [22.00, 26.43] | 23.71 [21.86, 26.43] | 23.86 [22.29, 26.43] |
| Subcutaneous injection | 0.75 mg, n (%) | 626 (16.4) | 134 (18.2) | 492 (16.0) | 304 (17.6) | 322 (15.5) |
|  | 1.50 mg, n (%) | 2018 (52.9) | 368 (49.9) | 1650 (53.7) | 902 (52.2) | 1116 (53.6) |
|  | 3.00 mg, n (%) | 123 (3.2) | 25 (3.4) | 98 (3.2) | 57 (3.3) | 66 (3.2) |
|  | 4.50 mg, n (%) | 29 (0.8) | 9 (1.2) | 20 (0.7) | 13 (0.8) | 16 (0.8) |
| **8^th^ prescription, n (%)** | | 2689 (70.5) | 517 (70.1) | 2172 (70.7) | 1227 (71.0) | 1462 (70.2) |
| Time since Rx 1, weeks, median [IQR] | | 27.86 [26.00, 30.86] | 27.86 [26.14, 31.00] | 27.86 [26.00, 30.71] | 27.71 [25.93, 30.86] | 27.86 [26.14, 30.71] |
| Subcutaneous injection | 0.75 mg, n (%) | 581 (15.2) | 121 (16.4) | 460 (15.0) | 284 (16.4) | 297 (14.3) |
|  | 1.50 mg, n (%) | 1947 (51.1) | 360 (48.8) | 1587 (51.6) | 872 (50.4) | 1075 (51.6) |
|  | 3.00 mg, n (%) | 129 (3.4) | 26 (3.5) | 103 (3.4) | 56 (3.2) | 73 (3.5) |
|  | 4.50 mg, n (%) | 32 (0.8) | 10 (1.4) | 22 (0.7) | 15 (0.9) | 17 (0.8) |
| **9^th^ prescription, n (%)** | | 2587 (67.9) | 497 (67.3) | 2090 (68.0) | 1188 (68.7) | 1399 (67.2) |
| Time since Rx 1, weeks, median [IQR] | | 31.86 [30.00, 34.86] | 31.86 [30.00, 35.14] | 31.86 [30.00, 34.86] | 31.64 [29.82, 34.86] | 32.00 [30.14, 35.00] |
| Subcutaneous injection | 0.75 mg, n (%) | 547 (14.3) | 114 (15.4) | 433 (14.1) | 266 (15.4) | 281 (13.5) |
|  | 1.50 mg, n (%) | 1861 (48.8) | 342 (46.3) | 1519 (49.4) | 842 (48.7) | 1019 (48.9) |
|  | 3.00 mg, n (%) | 149 (3.9) | 31 (4.2) | 118 (3.8) | 67 (3.9) | 82 (3.9) |
|  | 4.50 mg, n (%) | 30 (0.8) | 10 (1.4) | 20 (0.7) | 13 (0.8) | 17 (0.8) |
| **10^th^ prescription, n (%)** | | 2474 (64.9) | 468 (63.4) | 2006 (65.3) | 1130 (65.4) | 1344 (64.5) |
| Time since Rx 1, weeks, median [IQR] | | 35.86 [33.89, 39.00] | 35.86 [34.00, 39.46] | 35.86 [33.86, 38.86] | 35.86 [33.71, 38.86] | 35.93 [34.00, 39.00] |
| Subcutaneous injection | 0.75 mg, n (%) | 505 (13.2) | 102 (13.8) | 403 (13.1) | 242 (14.0) | 263 (12.6) |
|  | 1.50 mg, n (%) | 1769 (46.4) | 326 (44.2) | 1443 (46.9) | 794 (45.9) | 975 (46.8) |
|  | 3.00 mg, n (%) | 165 (4.3) | 32 (4.3) | 133 (4.3) | 78 (4.5) | 87 (4.2) |
|  | 4.50 mg, n (%) | 35 (0.9) | 8 (1.1) | 27 (0.9) | 16 (0.9) | 19 (0.9) |

| Table S15. Characteristics linked to glucagon-like peptide-1 (GLP-1) receptor agonist therapy discontinuation within 1-year. | | | | |
| --- | --- | --- | --- | --- |
| **Characteristics of interest** | **Hazard ratio (95% CI)** | **p value** | **Hazard ratio (95% CI)** | **p value** |
| **Individuals included** | **GLP-1RA initiators with a recent BMI measurement (n=6245; 76.2%)** | | | |
| **Definition of discontinuation** | Discontinuation of GLP-1RA therapy was defined as a gap of >90-days, while switching between GLP-1RA agents was not considered as discontinuation. | | Discontinuation of GLP-1RA therapy was defined as either a gap of >90-days between subsequent prescriptions or a switch to a different GLP-1RA agent. | |
| **BMI group** |  | |  | |
| <30 kg/m^2^ | Reference | NA | Reference | NA |
| 30-<35 kg/m^2^ | 0.77 (0.70-0.85) | <0.001 | 0.78 (0.71-0.86) | <0.001 |
| ≥35 kg/m^2^ | 0.67 (0.61-0.74) | <0.001 | 0.69 (0.63-0.76) | <0.001 |
| **Sex** |  | |  | |
| Female | Reference | NA | Reference | NA |
| Male | 1.07 (0.99-1.16) | 0.089 | 1.07 (0.99-1.16) | 0.0814 |
| **Cardiovascular disease** |  | |  | |
| No | Reference | NA | Reference | NA |
| Yes | 0.92 (0.84-1.02) | 0.106 | 0.93 (0.84-1.02) | 0.108 |
| **GLP-1RA agent initiated** |  | |  | |
| Dulaglutide | Reference | NA | Reference | NA |
| Semaglutide | 1.51 (1.40-1.64) | <0.001 | 1.48 (1.37-1.6) | <0.001 |
| Liraglutide | 1.50 (1.34-1.69) | <0.001 | 1.72 (1.55-1.92) | <0.001 |
| Exenatide | 1.39 (1.12-1.71) | <0.01 | 1.60 (1.31-1.96) | <0.001 |
| Lixisenatide | 2.30 (1.48-3.59) | <0.001 | 2.73 (1.78-4.18) | <0.001 |
| **Individuals included** | **Semaglutide initiators with a recent BMI measurement (n=2382; 75.1%)** | | **Dulaglutide initiators with a recent BMI measurement (n=2926; 76.8%)** | |
| **Definition of discontinuation** | Discontinuation of GLP-1RA therapy was defined as either a gap of >90-days between subsequent prescriptions or a switch to a different GLP-1RA agent. | | Discontinuation of GLP-1RA therapy was defined as either a gap of >90-days between subsequent prescriptions or a switch to a different GLP-1RA agent. | |
| **Characteristics of interest** | **Hazard ratio (95% CI)** | **p value** | **Hazard ratio (95% CI)** | **p value** |
| **BMI group** |  | |  | |
| <30 kg/m^2^ | Reference | NA | Reference | NA |
| 30-<35 kg/m^2^ | 0.82 (0.71-0.95) | 0.00715 | 0.77 (0.66-0.89) | <0.001 |
| ≥35 kg/m^2^ | 0.74 (0.64-0.86) | <0.001 | 0.65 (0.56-0.76) | <0.001 |
| **Sex** |  | |  | |
| Female | Reference | NA | Reference | NA |
| Male | 1.06 (0.94-1.19) | 0.355 | 1.08 (0.96-1.22) | 0.211 |
| **Cardiovascular disease** |  | |  | |
| No | Reference | NA | Reference | NA |
| Yes | 1.01 (0.87-1.16) | 0.945 | 0.9 (0.78-1.05) | 0.178 |
| **Formulation** |  | |  | |
| Subcutaneous injection | Reference | NA | NA | NA |
| Oral tablet | 1.27 (1.12-1.45) | <0.001 | NA | NA |
| Abbreviations: GLP-1RA glucagon-like peptide-1 receptor agonist, BMI body-mass index. Associations of index GLP-1RA initiated, BMI category, CVD history, and sex with the risk of GLP-1RA discontinuation were assessed using cause-specific Cox proportional hazards regression analyses, with censoring at the date of death or transfer out of the GP practice. The multivariable Cox model was restricted to the 6245 (76.2%) individuals with a recent BMI measurement and was adjusted for variables specified in Supplementary Table S3. | | | | |

# SUPPLEMENTARY FIGURES

| **** |
| --- |
| Figure S1. Cohort construction of individuals initiating glucagon-like peptide-1 (GLP-1) receptor agonists using the IQVIA Medical Research Data (IMRD) incorporating data from THIN, A Cegedim Database. |

# SUPPLEMENTARY REFERENCES

1. Novo Nordisk A/S - SPC MHRA products. Liraglutide - Summary of Product Characteristics (SPC). https://mhraproducts4853.blob.core.windows.net/docs/ae9081cc57d330932293bacd4759d163e3c0f645 (06-Sept-2024).

2. NHS dm+d browser. Actual Medicinal Product pack (AMPP) - Lyxumia. https://dmd-browser.nhsbsa.nhs.uk/ampp/view/98700?ref= (06-Sept-2024).

3. Aventis Pharma Limited. Lixisenatide + Insulin glargine - Summary of Product Characteristics (SPC). https://mhraproducts4853.blob.core.windows.net/docs/ef68271956f8a4b40c6e73926f97ca606eca8f55 (06-Sept-2024).

4. NHS dm+d browser. Actual Medicinal Product pack (AMPP) - Eperzan. https://dmd-browser.nhsbsa.nhs.uk/ampp/view/143715?ref=YW1wTmFtZT1BbGJpZ2x1dGlkZSsmc2VhcmNoVHlwZT1BTVAmc2hvd0ludmFsaWRJdGVtcz1mYWxzZSZoaWRlUGFyYWxsZWxJbXBvcnQ9ZmFsc2UmaGlkZVNwZWNpYWxPcmRlcj1mYWxzZSZoaWRlRGlzY29udGludWVkSXRlbXM9ZmFsc2U%3D (06-Sept-2024).

5. Inker, L. A. *et al.* New Creatinine- and Cystatin C-Based Equations to Estimate GFR without Race. *N Engl J Med* **385**, 1737–1749 (2021).
